# NLRP3 Inflammasome Mediates Silica-induced Lung Epithelial Injury and Aberrant Regeneration in Lung Stem/Progenitor Cell-derived Organotypic Models

**DOI:** 10.7150/ijbs.80605

**Published:** 2023-03-21

**Authors:** Hong Zhou, Qun Zhang, Wen Huang, Shulan Zhou, Yanli Wang, Xiaoning Zeng, Hong Wang, Weiping Xie, Hui Kong

**Affiliations:** Department of Pulmonary & Critical Care Medicine, The First Affiliated Hospital of Nanjing Medical University, Nanjing, Jiangsu 210029, P.R. China

**Keywords:** NLRP3 inflammasome, Lung stem/progenitor cells (LSPCs), Organoids, Repair, Regeneration

## Abstract

Silica-induced lung epithelial injury and fibrosis are vital pathogeneses of silicosis. Although the NOD-like receptor protein 3 (NLRP3) inflammasome contributes to silica-induced chronic lung inflammation, its role in epithelial injury and regeneration remains unclear. Here, using mouse lung stem/progenitor cell-derived organotypic systems, including 2D air-liquid interface and 3D organoid cultures, we investigated the effects of the NLRP3 inflammasome on airway epithelial phenotype and function, cellular injury and regeneration, and the potential mechanisms. Our data showed that silica-induced NLRP3 inflammasome activation disrupted the epithelial architecture, impaired mucociliary clearance, induced cellular hyperplasia and the epithelial-mesenchymal transition in 2D culture, and inhibited organoid development in 3D system. Moreover, abnormal expression of the stem/progenitor cell markers SOX2 and SOX9 was observed in the 2D and 3D organotypic models after sustained silica stimulation. Notably, these silica-induced structural and functional abnormalities were ameliorated by MCC950, a selective NLRP3 inflammasome inhibitor. Further studies indicated that the NF-κB, Shh-Gli and Wnt/β-catenin pathways were involved in NLRP3 inflammasome-mediated abnormal differentiation and dysfunction of the airway epithelium. Thus, prolonged NLRP3 inflammasome activation caused injury and aberrant lung epithelial regeneration, suggesting that the NLRP3 inflammasome is a pivotal target for regulating tissue repair in chronic inflammatory lung diseases.

## Introduction

The occurrence of chronic inflammation in the lung due to various insults is a pivotal etiology for the development of chronic obstructive pulmonary disease (COPD), corona virus disease 2019 (COVID-19), idiopathic pulmonary fibrosis (IPF), occupational pulmonary disease, and other conditions 1-4. Silicosis, an incurable and progressive pulmonary disease, is characterized by persistent inflammation and irreversible fibrosis caused by the inhalation of silica particles 5, 6. Accumulating evidence supports the observation that sustained damage to the distal airway epithelium due to silica is the dominant cause of airway structure remodeling and lung fibrosis in silicosis 7. Histologically, terminal bronchioles are particularly susceptible to silica particles, which cause mucous plugging 8, epithelial dysfunction 9, 10 and ultimately peribronchiolar fibrosis, bronchiolar obstruction or obliteration 11, 12.

Regarding pulmonary fibrotic diseases, previous studies have reinforced the importance of macrophages, fibroblasts and epithelial cells in maintaining the structural and functional integrity of the lung 13-16. However, recent studies suggest that lung stem/progenitor cells (LSPCs) play an important role in lung tissue injury, repair and regeneration 17, 18. Increasing experimental evidence has revealed that epithelial progenitor cell injury and aberrant repair lead to fibrosis and bronchiolitis obliterans-like conditions 19. With repeated exposure to silica particles, LSPC proliferation and differentiation are impaired during the development of silicosis 10. In IPF, ongoing injurious stimuli induce an aberrant environment that drives LSPCs away from terminal differentiation and initiates a profibrotic cascade 20. In addition, distal airway stem/progenitor cells could ameliorate bleomycin-induced pulmonary fibrosis by promoting lung regeneration 21.

The NLRP3 inflammasome is a widely distributed cytosolic multiprotein complex comprising NOD-like receptor protein 3 (NLRP3), apoptosis-associated speck-like protein containing a CARD domain (ASC), and pro-Caspase-1. The assembled NLRP3 inflammasome promotes pro-Caspase-1 maturation and converts pro‐interleukin‐1β (IL‐1β) and pro‐IL‐18 into their bioactive forms 22, 23. To date, the NLRP3 inflammasome has been reported to play a central role in several inflammatory respiratory diseases, such as asthma 24, sarcoidosis 25, cystic fibrosis 26, acute lung injury 27 and COVID-19 28, 29. In addition, silica is a well-recognized activator of the NLRP3 inflammasome, which induces inflammatory damage and mesenchymal transition of airway epithelial cells, thereafter promoting pulmonary fibrosis in silicosis 30, 31. However, whether silica-induced NLRP3 inflammasome activation mediates LSPC injury, repair and regeneration remains unclear.

Lung organotypic models are stem/progenitor cell-derived and self-organizing structures. They simulate the morphogenesis, structures and functions of the respiratory system *in vivo*, providing a versatile and reliable *in vitro* culture system to study lung development and regenerative medicine, model diseases and screen drugs 32, 33. During the past decade, lung organotypic models, including 2D air-liquid interface (ALI) and 3D organoid cultures, have become remarkable platforms to investigate the differentiation and repair of LSPCs, morphogenesis and function of ciliated epithelium, as well as mucus production and airway clearance 34, 35. Recently, important advances in illustrating the mechanisms of COVID-19 have been reported using organoid platforms 36, 37. In this study, we employed mouse LSPC-derived lung epithelial organotypic models (including ALI and organoid cultures) to unveil the effects and mechanisms of lung epithelial injury and regeneration in response to sustained silica stimulation. We discovered the critical role of NLRP3 inflammasome activation in enhancing silica-induced lung epithelial injury and aberrant repair, providing a promising therapeutic target for inhalable particle-related lung injuries and diseases.

## Results

### Silica-induced NLRP3 inflammasome activation caused disorganization and pyroptosis of the LSPC-derived airway epithelium in the ALI model

**Figure 1:**
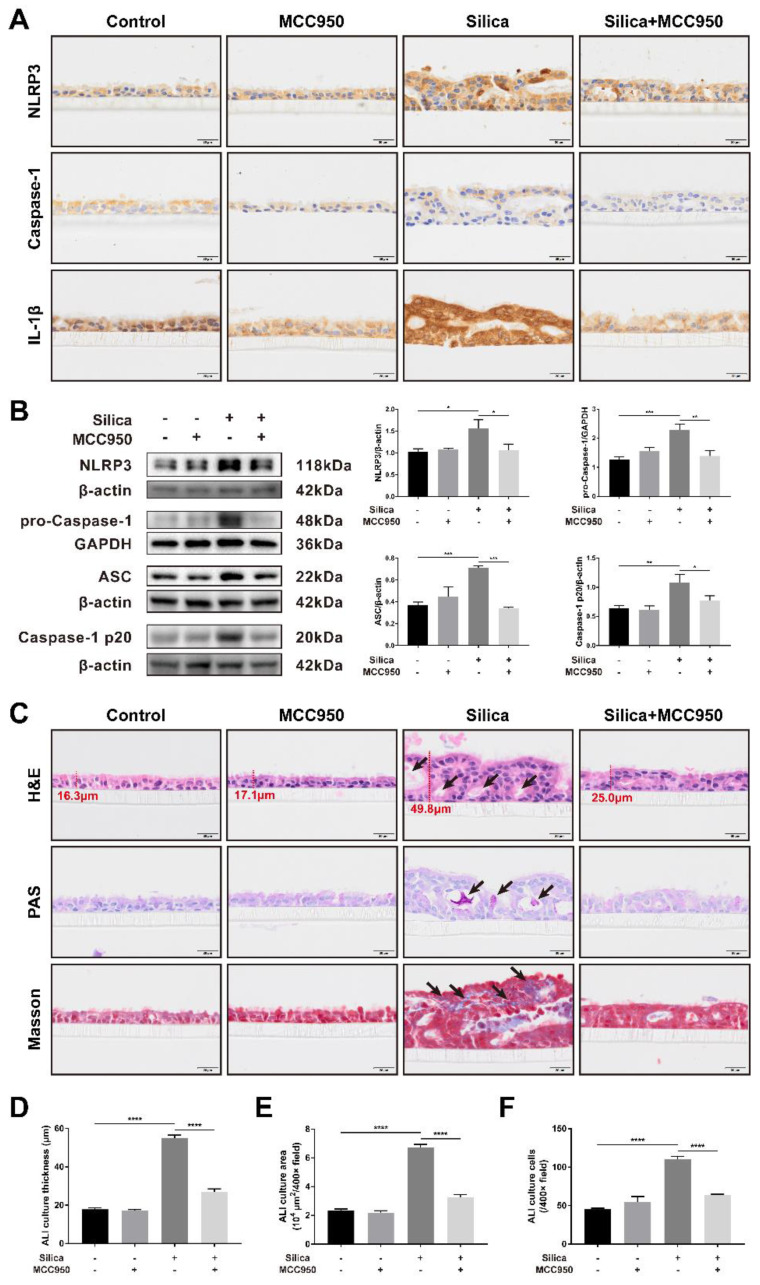
** Effects of silica-induced NLRP3 inflammasome activation on the disorganization of the LSPC-derived airway epithelium in the ALI model. (A)** Representative photographs of immunohistochemistry for NLRP3, Caspase-1 and IL-1β staining in the z-stack of ALI cultures. Scale bar, 20 μm. **(B)** Representative images and western blot analysis of NLRP3, pro-Caspase-1, ASC and Caspase-1 p20 in ALI cultures. MCC950 markedly decreased silica-induced NLRP3 inflammasome activation in the LSPC-derived airway epithelium (n=3-4). Data are presented as the mean ± standard error of the mean (SEM): *P<0.05, **P<0.01, ***P<0.001. **(C)** Representative images of H&E, PAS and Masson's trichrome staining in the z-stack of ALI cultures. Large cavities (H&E, black arrows), mucus overproduction (PAS, black arrows) and collagen fiber accumulation (Masson, black arrows). Scale bar, 20 μm. **(D, E & F)** MCC950 markedly reduced the thickness of silica-induced airway epithelium **(D)**, area **(E)** and cell numbers **(F)** under high magnification (×400) (n=3). Data are expressed as the mean ± SEM: ****P<0.0001.

An airway epithelium-like structure from the lungosphere-dissociated LSPCs was successfully generated in our ALI culture system, as indicated by the formation of pseudostratified columnar ciliated epithelium-containing MUC5AC^+^ goblet cells (Figures S1 and S2). As shown in Figures 1A and 1B, both the immunohistochemistry and western blot results for the components and products of the NLRP3 inflammasome (including NLRP3, pro-Caspase-1, ASC, Caspase-1 p20 and IL-1β) confirmed that sustained silica stimulation resulted in significant activation of the NLRP3 inflammasome in the LSPC-differentiated airway epithelium, which was blocked by MCC950, a selective NLRP3 inflammasome inhibitor. Notably, routine hematoxylin and eosin (H&E), periodic acid-Schiff (PAS), and Masson's trichrome staining showed that sustained stimulation with silica for 28 days led to thickened and disorganized LSPC-differentiated airway epithelium that exhibited irregular surfaces, large cavities, overproduction of mucus, and accumulation of collagen fibers (Figures 1C-F). A single treatment with MCC950 alone had no significant effects on the generation of the airway epithelium, while it prevented the epithelial disorganization induced by silica (Figure 1C).

**Figure 2:**
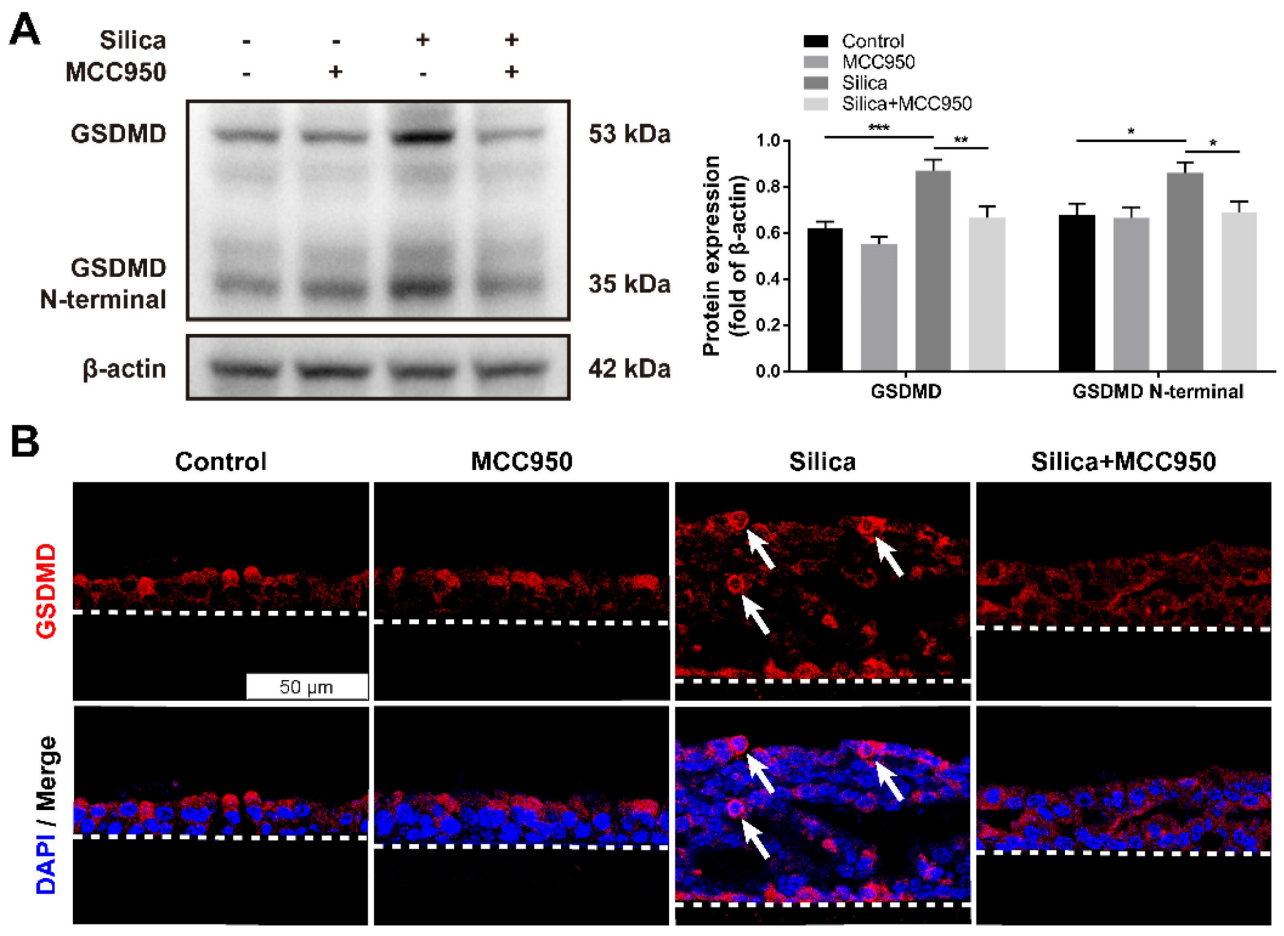
** Effects of silica-induced NLRP3 inflammasome activation on pyroptosis of LSPC-derived airway epithelial cells in the ALI model. (A)** Representative images and western blot analysis of GSDMD and GSDMD N-terminal domains in ALI cultures. MCC950 attenuated silica-induced pyroptosis with the downregulated expression of GSDMD and GSDMD N-terminal (n=5). Data are shown as the mean ± SEM: *P<0.05, **P<0.01, ***P<0.001. **(B)** Representative photographs of immunofluorescence staining for GSDMD in the z-stack of ALI cultures. White arrows indicate active GSDMD with an intense signal on the cell membrane. Scale bar, 50 μm.

Pyroptosis is a specific programmed cell death mediated by the activated NLRP3 inflammasome that is involved in various respiratory diseases, including pulmonary fibrosis 30, 38. Once activated, the NLRP3 inflammasome downstream of Caspase-1 cleaves cytoplasmic Gasdermin D (GSDMD) to release its N-terminal domain, thereafter forming membrane pores to induce pyroptotic cell death 39. As shown in the western blot analysis, compared to that of the control group, silica treatment significantly increased the levels of GSDMD and the GSDMD N-terminal compared with the control group, changes that were reversed by the NLRP3 inflammasome inhibitor MCC950 (Figure 2A). Consistently, the immunofluorescence staining showed that sustained silica stimulation resulted in a greater number of membrane-distributed GSDMD^+^ cells in LSPC-derived airway epithelial cells in the ALI model, which was inhibited by MCC950 (Figure 2B).

### NLRP3 inflammasome activation led to abnormal mucociliary differentiation, multiciliogenesis and mucus hypersecretion from the LSPC-derived airway epithelium

**Figure 3:**
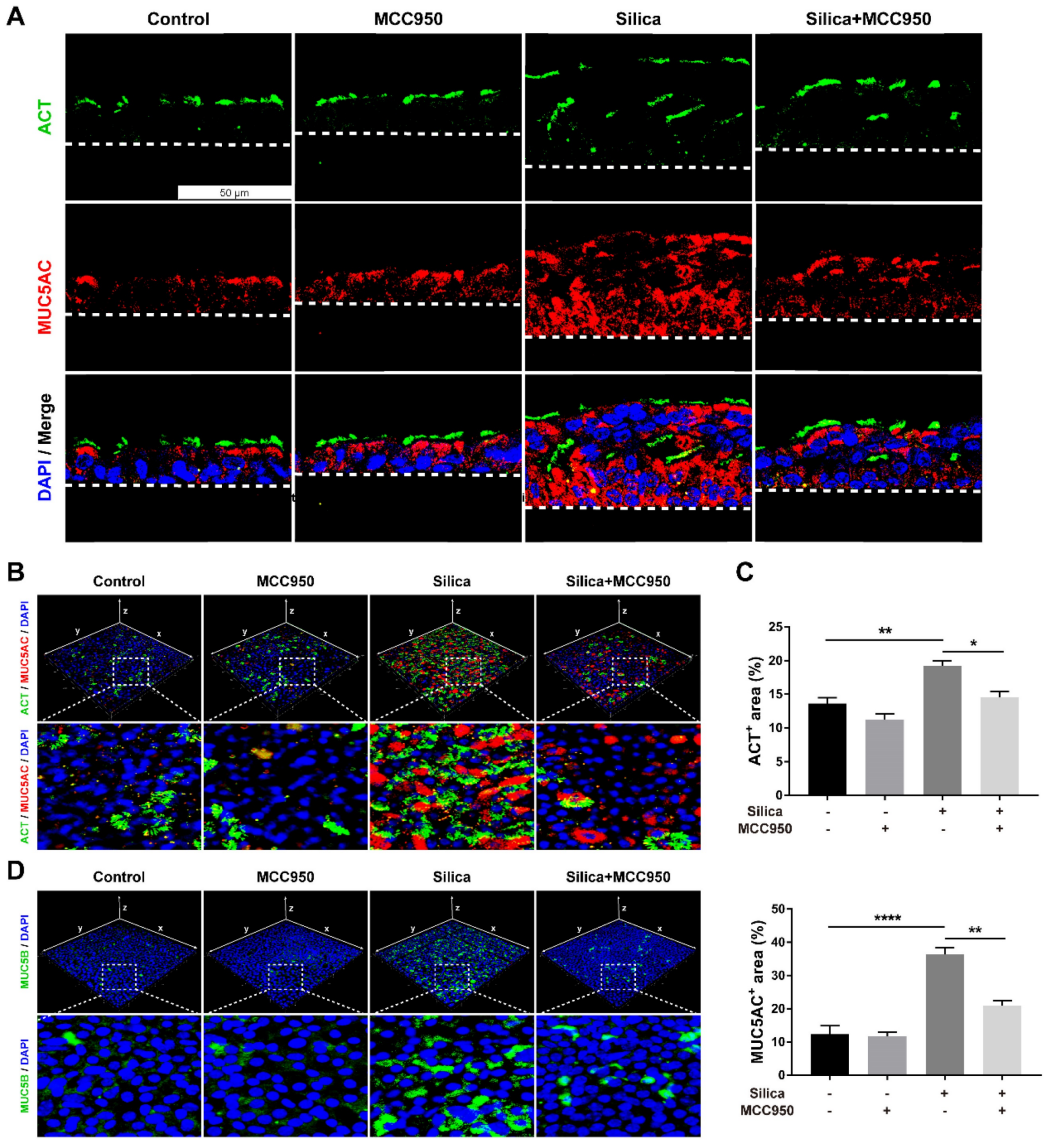
** Effects of silica on mucociliary differentiation, multiciliogenesis and hypersecretion of the LSPC-derived airway epithelium in the ALI model. (A)** Representative immunofluorescent images of ALI cultures in the z-stack. ACT (green): cilia marker, MUC5AC (red): mucus-producing goblet cell marker. Scale bar, 50 μm. **(B)** 3D reconstruction (xyz plane) of ACT (green) and MUC5AC (red) staining of ALI cultures. **(C)** MCC950 treatment decreased the percentage of ACT-positive or MUC5AC-positive stained area in the x-y plane of ALI cultures (n=3). Data are shown as the mean ± SEM: *P<0.05, **P<0.01, ****P<0.0001. **(D)** 3D reconstruction (xyz plane) of MUC5B (green) staining of ALI cultures.

Since routine histological staining found that silica treatment resulted in abnormal architecture of the LSPC-derived airway epithelium, the cellular phenotype and function of epithelial cells were investigated further. Immunofluorescence staining showed that silica increased the number of acetylated tubulin-positive (ACT^+^) ciliated cells and mucin 5AC-positive (MUC5AC^+^) mucus-producing goblet cells that were distributed in the LSPC-derived airway epithelium (Figures 3A-C). Moreover, compared to the mainly apical distribution in the control group, MUC5AC^+^ goblet cells were spread in the whole layer, while ACT^+^ ciliated cells were distributed in either the surface or inner layer of the LSPC-derived airway epithelium stimulated by silica. These multiple layers of ACT^+^ ciliated cells suggested that silica treatment resulted in epithelial layer stacking in the LSPC-derived airway epithelium (Figures 1A, 1C and 3A). A 3D reconstruction of the immunofluorescence results was obtained, which showed that mucin 5B (MUC5B), another gel-forming mucin associated with airway hypersecretion 40, was also overexpressed in the airway epithelium in response to silica (Figure 3D). However, these abnormalities in mucociliary differentiation, multiciliogenesis and mucus hypersecretion induced by silica were significantly suppressed by the NLRP3 inflammasome inhibitor MCC950 (Figures 3A-D).

### NLRP3 inflammasome activation resulted in ciliary dysplasia and dyskinesia in the LSPC-derived airway epithelium

**Figure 4:**
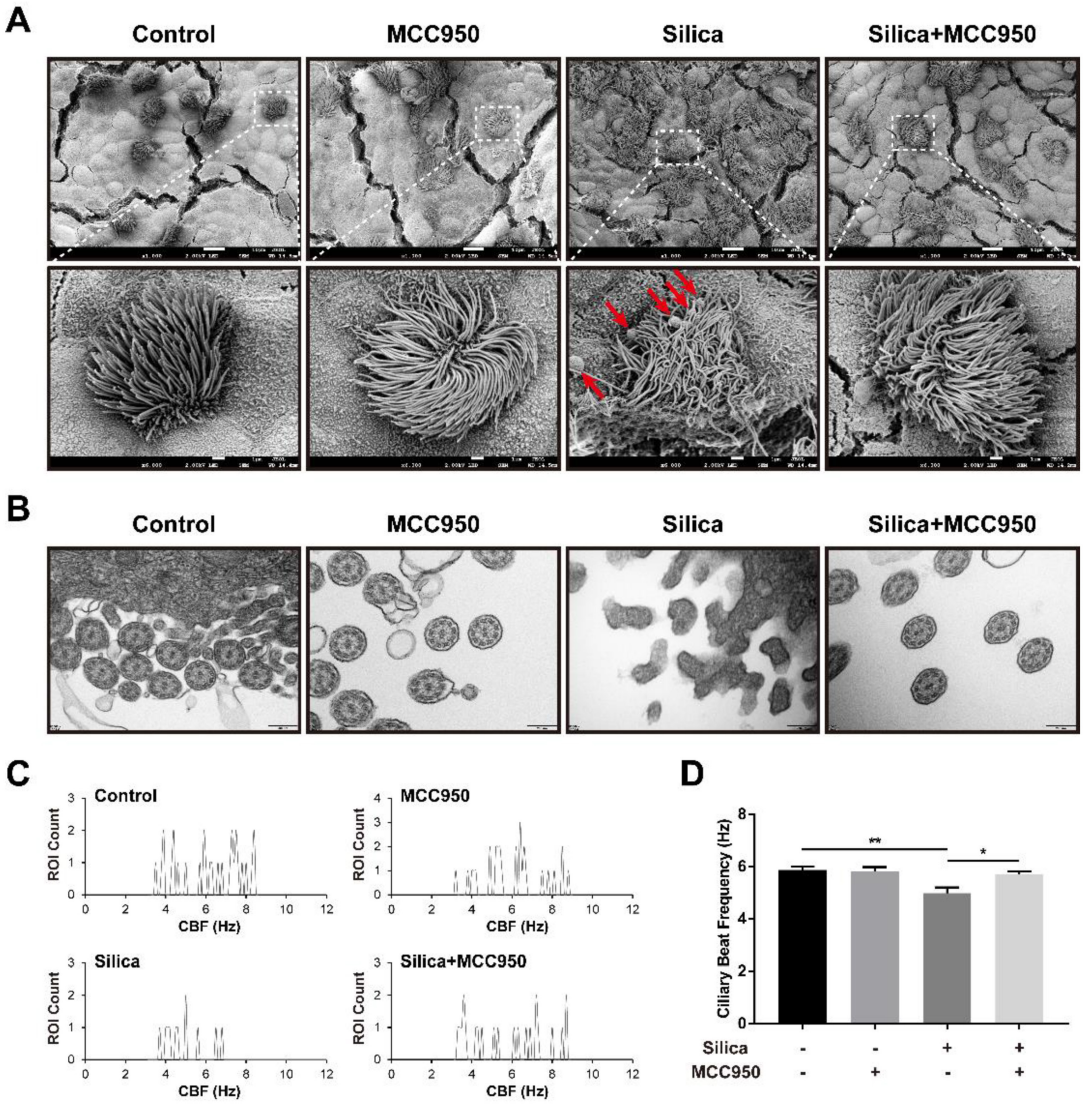
** Effects of silica on ciliary dysplasia and dyskinesia in the LSPC-derived airway epithelium in the ALI model. (A)** Collapsed and entangled cilia and mucus globules (red arrows) were observed on the apical surface of the airway epithelium in the silica-treated group by scanning electron microscopy (SEM). MCC950 treatment partially alleviated the silica-induced distortion of cilia architecture and accumulation of large mucus globules. Scale bars, 10 μm and 1 μm (magnified picture). **(B)** Transverse sections of cilia on the apical surface of the airway epithelium revealed that the normal ultrastructure of “9*2+2” microtubules was damaged in the silica-treated group according to transmission electron microscopy (TEM). MCC950 treatment partially ameliorated the fusion of silica-induced cilia with the plasma membrane. Scale bar, 200 nm. **(C)** Representative ROI count results obtained from the ciliaFA plugin in ImageJ. **(D)** Selective NLRP3 inflammasome inhibitor MCC950 improved silica-induced ciliary dyskinesia as determined by measuring the ciliary beat frequency (CBF) (n=3). Data are presented as the mean ± SEM: *P<0.05, **P<0.01.

Based on our findings of elevated numbers of ciliated cells and goblet cells by treatment with silica, we further studied the ultrastructural abnormalities of cilia in the LSPC-derived airway epithelium using scanning electron microscopy (SEM) and transmission electron microscopy (TEM). In the silica-treated group, the SEM analysis showed that cilia on the surface of the LSPC-derived airway epithelium were collapsed and entangled with an irregular length compared to those in the control group and MCC950 groups (Figure 4A). Additionally, the overproduction of mucus induced by silica was also confirmed by SEM, as indicated by the greater accumulation of mucus globules on the apical surface of the epithelium (Figure 4A, red arrows). Moreover, TEM revealed that silica treatment disrupted the formation of a normal “9*2+2” microtubule organization (the nine doublets and a central pair of singlet microtubules with dynein arms) in the cilia structure 41, 42 and led to cilia fusion with the plasma membrane (Figure 4B). These silica-induced pathological changes in cilia on the surface of the LSPC-derived airway epithelium observed by electron microscopy were prevented by the selective NLRP3 inflammasome inhibitor MCC950. These structural abnormalities of cilia suggested that cilia motility was dysfunctional. Furthermore, the ciliary beat frequency (CBF) of cilia on the LSPC-derived airway epithelium was analyzed by ciliaFA software 41, 43. As shown in Figures 4C and 4D, CBF was significantly inhibited by treatment with silica in the ALI model, which was reversed by the NLRP3 inflammasome inhibitor MCC950. Thus, NLRP3 inflammasome activation not only disrupted the normal structure but also impaired the mucus clearance function of cilia in the LSPC-derived airway epithelium.

### NLRP3 inflammasome activation enhanced cell proliferation and the epithelial-mesenchymal transition (EMT) in the LSPC-derived airway epithelium

**Figure 5:**
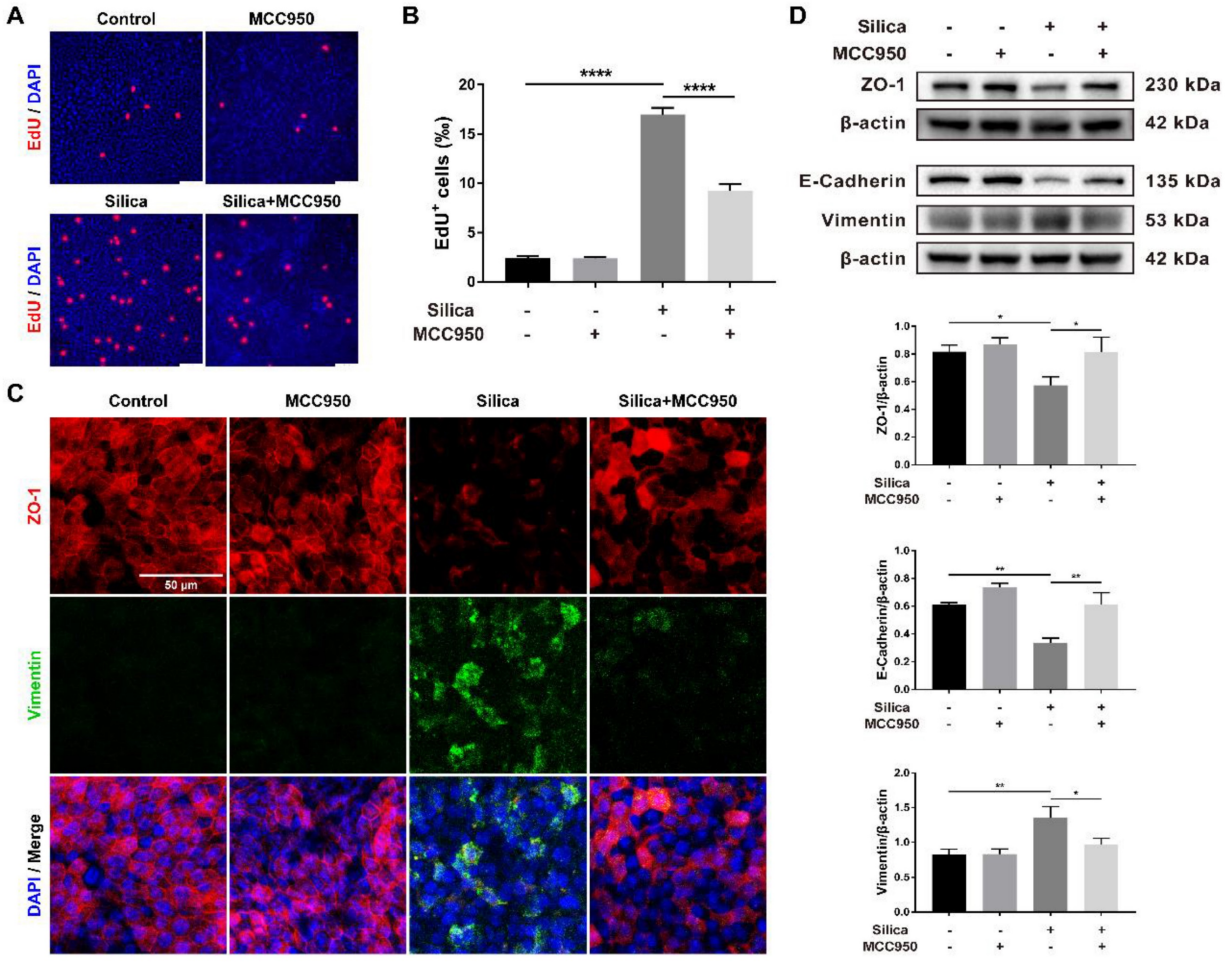
** Effects of silica-induced NLRP3 inflammasome activation on cell proliferation and epithelial-mesenchymal transition (EMT) of the LSPC-derived airway epithelium in the ALI model. (A)** Representative photographs of the EdU incorporation assay of ALI cultures in the x-y plane. Scale bar, 50 μm. **(B)** Quantification of EdU-positive cells in the x-y plane (n=3). The proportion of silica-induced EdU-positive cells was decreased by the NLRP3 inflammasome inhibitor MCC950. Data are presented as the mean ± SEM: ****P<0.0001. **(C)** Representative images of positive immunostaining of ZO-1 (red) and Vimentin (green) in the x-y plane. Scale bar, 50 μm. **(D)** Representative photographs and western blot analysis of epithelial markers (ZO-1 and E-Cadherin) and a mesenchymal marker (Vimentin) in ALI cultures. MCC950 treatment alleviated silica-induced EMT with upregulation of ZO-1 (n=4) and E-Cadherin (n=3) and downregulation of Vimentin (n=5). The data are expressed as the mean ± SEM: *P<0.05, **P<0.01.

Morphological studies showed that although silica-induced NLRP3 inflammasome activation enhanced pyroptotic cell death (Figures 2A and 2B), it thickened the LSPC-derived airway epithelium in the ALI model (Figures 1A, 1C, 1D and 3A). This result suggested that NLRP3 inflammasome activation might affect epithelial cell proliferation. As shown in Figures 5A and 5B, EdU staining confirmed that silica treatment significantly promoted cell proliferation in the LSPC-derived airway epithelium, which was inhibited by MCC950.

EMT is an important indicator for organ fibrosis and epithelial cell dysfunction 31, 44, 45. Our immunofluorescence study revealed that silica treatment decreased the expression of ZO-1 (an epithelial marker) while increasing the expression of Vimentin (a mesenchymal marker) in the LSPC-derived airway epithelium in ALI cultures (Figure 5C). This result was confirmed by the western blot analysis, which showed that silica treatment significantly downregulated the expression of ZO-1 and E-Cadherin (another epithelial marker) but upregulated the expression of Vimentin (Figure 5D). Although MCC950 alone had no significant effects on the expression of EMT markers, it reversed the silica-induced downregulation of epithelial markers and upregulation of mesenchymal markers (Figures 5C and 5D).

### NLRP3 inflammasome activation induced an ectopic distribution of LSPC-derived basal cells and high SOX2/SOX9 expression in the ALI model

**Figure 6:**
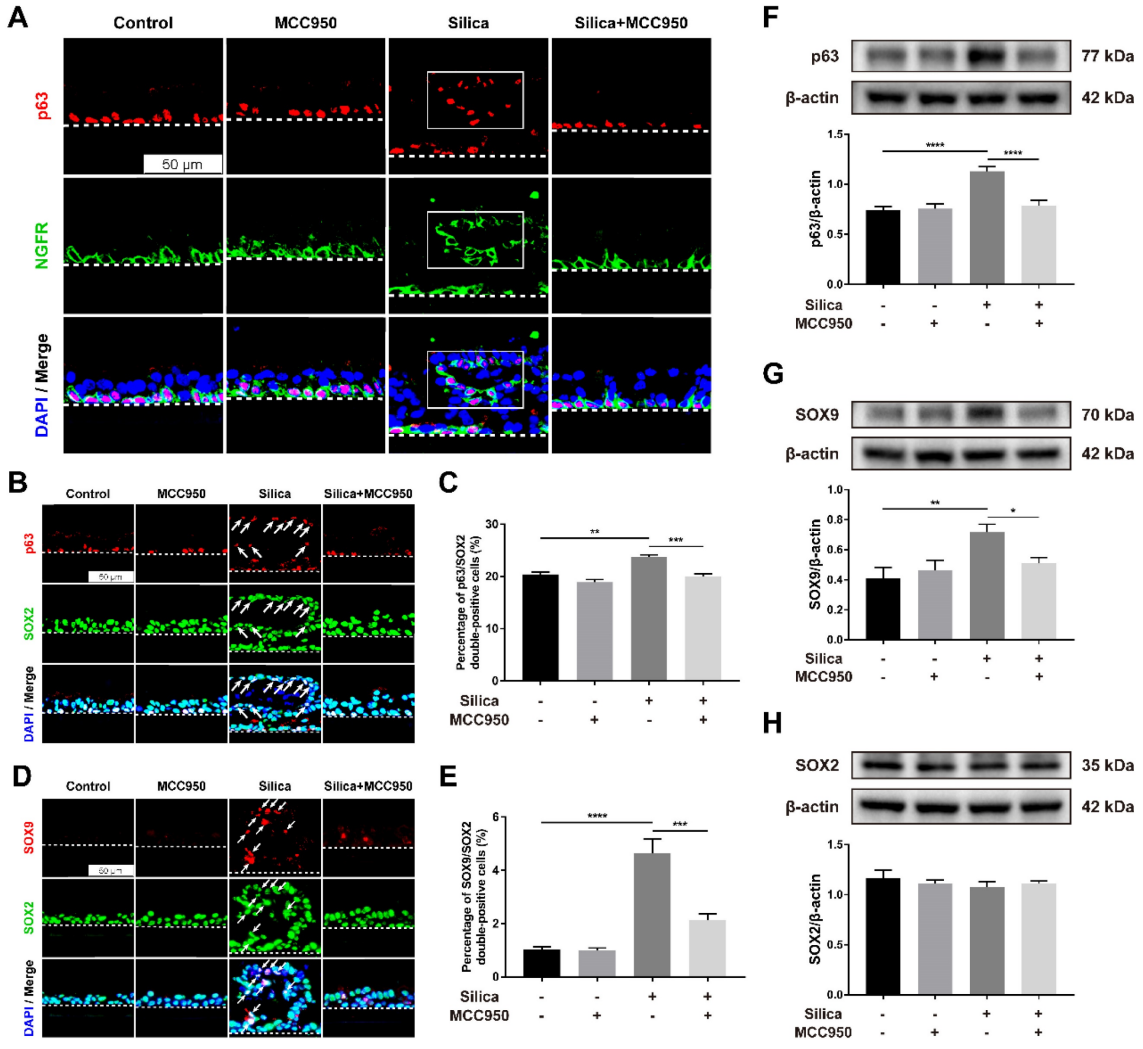
** Effects of silica-induced NLRP3 inflammasome activation on basal cell ectopic distribution and SOX2/SOX9 high expression in the ALI model. (A)** Representative images of immunofluorescence staining for p63 (red) and NGFR (green) in the z-stack of ALI cultures. The selected region (white box) indicated the silica-induced ectopic distribution of airway epithelial basal cells in the ALI model. Scale bar, 50 μm. **(B)** Representative images of immunofluorescence staining for p63 (red) and SOX2 (green) in the z-stack of ALI cultures. The white arrows indicate the silica-induced ectopic distribution of SOX2^+^ basal cells in ALI cultures. Scale bar, 50 μm. **(C)** Quantification of SOX2^+^ basal cells in the z-stack of ALI cultures (n=3). The percentage of SOX2^+^ basal cells in the silica-treated group was decreased by MCC950. The data are presented as the mean ± SEM: **P<0.01, ***P<0.001. **(D)** Representative images of immunofluorescence staining for SOX9 (red) and SOX2 (green) in the z-stack of ALI cultures. The white arrows indicate silica-induced SOX9/SOX2 double-positive cells in the ALI model. Scale bar, 50 μm. **(E)** Quantification of SOX9/SOX2 double-positive cells in the z-stack of ALI cultures (n=3). The percentage of double-positive cells in the silica-treated group was reduced by MCC950. The data are presented as the mean ± SEM: ***P<0.001, ****P<0.0001. **(F, G & H)** Representative photographs and western blot analysis of p63 **(F)**, SOX9 **(G)** and SOX2 **(H)** in ALI cultures. MCC950 markedly reduced silica-induced increases in the protein levels of p63 (n=5) and SOX9 (n=3), while no significant differences in SOX2 levels (n=3) were observed. The data are expressed as the mean ± SEM: *P<0.05, **P<0.01, ***P<0.001.

To further investigate the reparative behaviors of LSPCs in the ALI model in response to silica, the expression of important factors related to airway epithelial repair and regeneration, including p63, NGFR, SOX2, and SOX9, was examined 46-49. P63 and NGFR are markers of basal cells in the airway epithelium, and the ectopic presence of basal cell progenitor populations in the fibrotic areas has been observed in the bleomycin-induced injury murine model 50. In our control or MCC950 alone group, p63- and NGFR- coexpressing basal cells exhibited a restricted distribution on the basal layer of the LSPC-derived airway epithelium (Figure 6A). However, in the silica-treated group, basal cells were distributed not only on the basal layer but also on the upper layer, but their ectopic distribution was restored by treatment with the NLRP3 inflammasome inhibitor MCC950 (Figure 6A). Furthermore, SOX2 and SOX9 are two pluripotency-associated stem/progenitor cell transcription factors that drive the proliferation and differentiation of airway epithelial basal cells following injury 51-53. Our immunofluorescence results indicated that after stimulation with silica, most ectopic basal cells on the upper layer were positive for SOX2 (Figure 6B, white arrows), with a significantly increased proportion of SOX2^+^ basal cells in the LSPC-derived airway epithelium (Figure 6C). Moreover, increased coexpression of SOX9 and SOX2 was observed in the dysplastic epithelial cells after 28 days of sustained stimulation with silica compared to those in the control and MCC950 groups (Figures 6D and 6E). The western blot results showed consistently increased levels of p63 and SOX9 in the silica-treated group compared to the other groups (Figures 6F and 6G), while no significant differences in SOX2 levels were observed (Figure 6H). The aberrant reparative behaviors observed in the silica-stimulated group, including the ectopic distribution of basal cells and abnormal expression patterns of SOX2 and SOX9, were reversed by pharmacological inhibition of the NLRP3 inflammasome with MCC950 (Figures 6A-G).

### The NF-κB, Shh-Gli and Wnt/β-catenin pathways were involved in NLRP3 inflammasome activation-mediated dysfunction in the LSPC-derived airway epithelium

**Figure 7:**
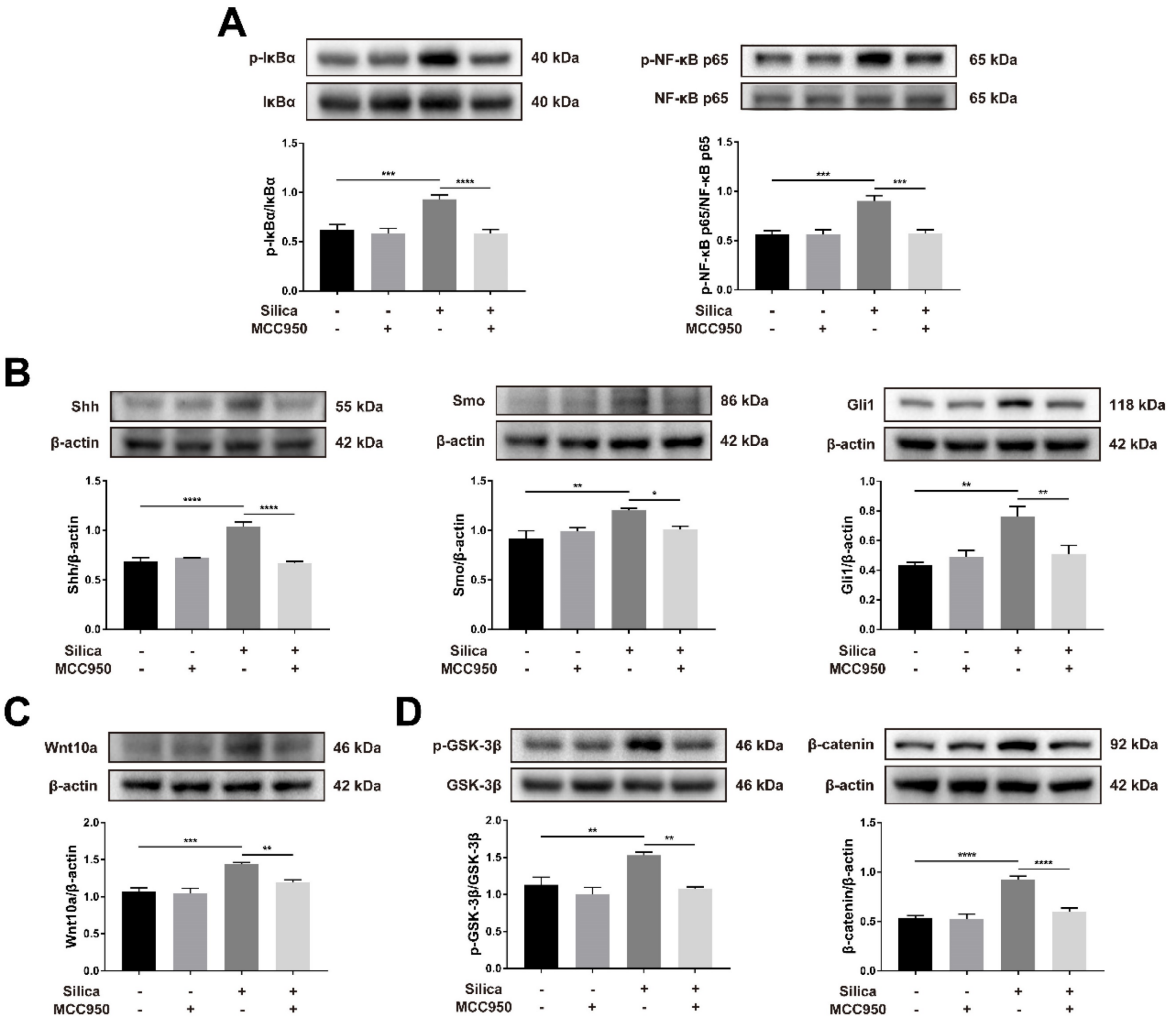
** Effects of silica-induced NLRP3 inflammasome activation on the NF-κB, Shh-Gli and Wnt/β-catenin pathways in ALI culture. (A)** Western blot analysis revealed that the phosphorylation-activation of IκBα (n=5) and NF-κB p65 (n=4) induced by silica was reversed by MCC950. Data are expressed as the mean ± SEM: ***P<0.001, ****P<0.0001. **(B)** Western blot analysis showed that the increased expression of Shh-Gli pathway-related proteins Shh (n=4), Smo (n=3) and Gli1 (n=3) induced by silica could be reversed by MCC950. The data are presented as the mean ± SEM: *P<0.05, **P<0.01, ****P<0.0001. **(C)** The high expression of Wnt10a (n=4) in the silica-treated group was measured by western blot and was reduced by MCC950. The data are expressed as the mean ± SEM: **P<0.01, ***P<0.001. **(D)** Western blot results showed that the silica-induced increased expression of the canonical Wnt/β-catenin signaling pathway-related molecules phosphorylated GSK-3β (n=3) and β-catenin (n=5) was reversed by MCC950. The data are presented as the mean ± SEM: **P<0.01, ****P<0.0001.

The nuclear factor-κB (NF-κB) signaling pathway is a classical inflammatory signaling pathway that closely interacts with the NLRP3 inflammasome to drive the production of inflammatory mediators 54, 55. Compared to the control, silica treatment significantly increased the phosphorylation-mediated activation of IκBα and NF-κB p65, and these effects were inhibited by MCC950 (Figure 7A). The hedgehog pathway is a vital signaling pathway for maintaining the homeostasis and regeneration of the epithelial barrier in response to the stress of inflammation 56. As shown in Figure 7B, sonic hedgehog (Shh, one major ligand of the hedgehog pathway) and its downstream transmembrane protein smoothened (SMO), as well as responsive transcription factor glioma-associated oncogene homolog 1 (Gli1), were significantly upregulated by silica treatment in ALI cultures. However, this silica-induced increase in Shh-Gli signal transduction was suppressed by the NLRP3 inflammasome inhibitor MCC950 (Figure 7B). The wingless-related integration site (Wnt) pathway is another important signaling pathway in lung development, repair and regeneration, and it affects cellular processes including proliferation, fate decisions, and apoptosis 57, 58. Wnt10a, a Wnt ligand involved in pulmonary fibrosis 59, 60, was upregulated in the silica-treated group compared to that in the control group (Figure 7C). Consistently, silica treatment led to the significant activation of Wnt downstream signaling including the phosphorylation of GSK-3β and accumulation of β-catenin in the LSPC-derived airway epithelium, which were blocked by the NLRP3 inflammasome inhibitor MCC950 (Figure 7D).

### Silica-induced NLRP3 inflammasome activation inhibited lung organoid development in 3D culture

**Figure 8:**
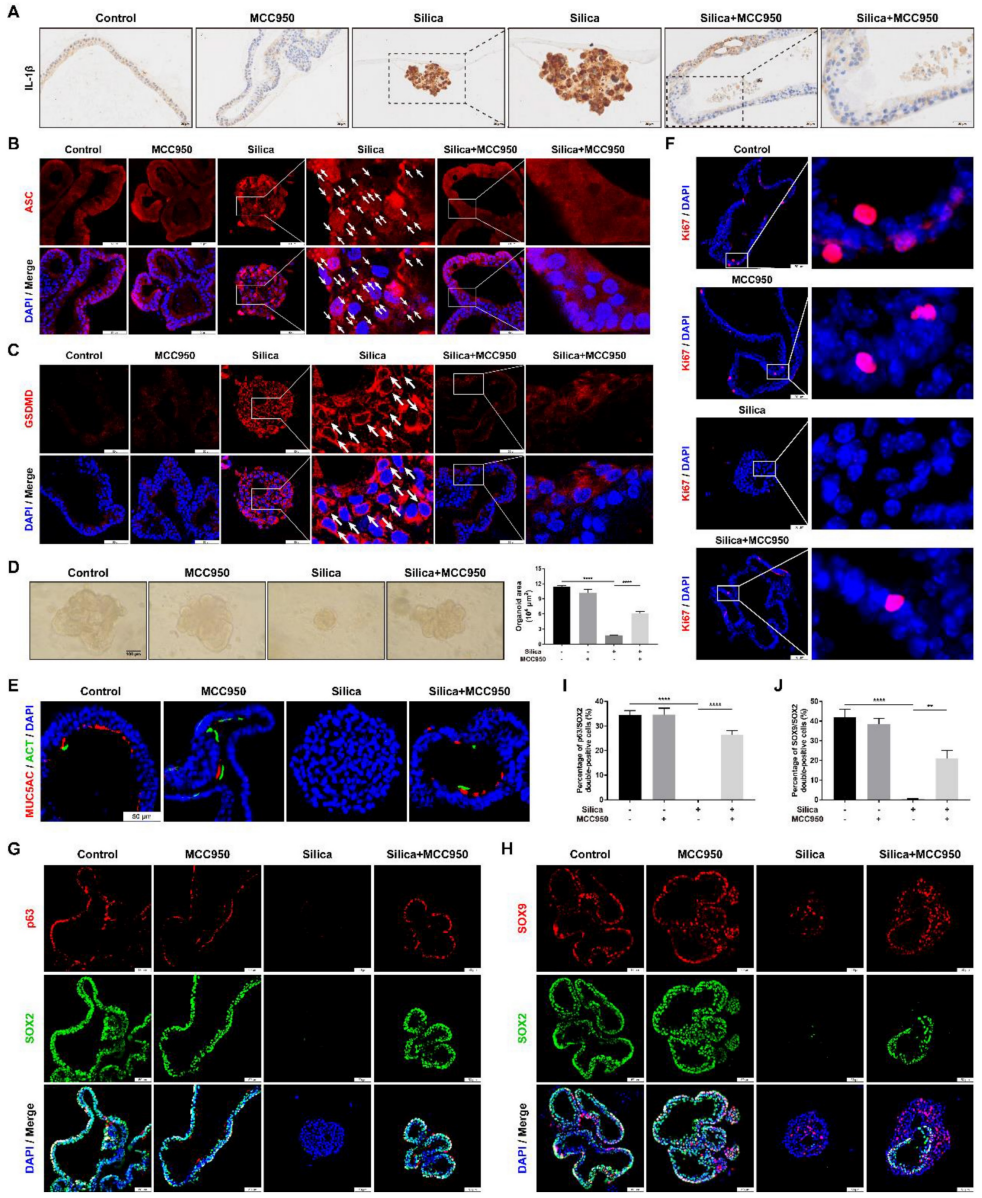
** Effects of silica-induced NLRP3 inflammasome activation on lung organoid development in 3D culture. (A)** Representative photographs of the immunohistochemistry for IL-1β staining of lung organoids. Scale bar, 20 μm. **(B)** Representative photographs of immunofluorescence staining for ASC specks in lung organoids. The white arrows indicate silica-induced ASC speck formation in the 3D system. Scale bar, 50 μm. **(C)** Representative images of immunofluorescence staining for GSDMD in organoids. The white arrows indicate the signal intensity of active GSDMD on the cell membrane in the silica-treated group. Scale bar, 50 μm. **(D)** Representative images of the lung organoids and quantification of the organoid area (n=3). Silica inhibited lung organoid development in the 3D culture, which was improved by MCC950 treatment. Scale bar, 100 μm. The data are presented as the mean ± SEM: ****P<0.0001. **(E, F, G & H)** Representative photographs of immunofluorescence staining for MUC5AC and ACT **(E)**, Ki67 **(F)**, p63 and SOX2 **(G)**, SOX9 and SOX2 **(H)** in the lung organoids. Scale bar, 50 μm. **(I & J)** Quantification of p63/SOX2 **(I)** and SOX9/SOX2 **(J)** double-positive cell numbers in the organoids (n=3). The data are expressed as the mean ± SEM: **P<0.01, ****P<0.0001.

Subsequently, the effects of sustained NLRP3 inflammasome activation on lung organoid development were investigated by using the 3D Matrigel culture system. In our 3D model, LSPC-derived lungospheres successfully generated lung organoids, as indicated by the formation of polarized airway epithelium containing cilia that pointed toward the lumen and PAS-positive mucus on the lumen-facing surface or in the lumen (Figures S1 and S3). Compared to the control and MCC950 groups, NLRP3 inflammasome activation in the silica-treated organoids was confirmed by strong signals for IL-1β immunostaining (Figure 8A), multiple cytoplasm-distributed ASC specks 61-63 (Figure 8B, white arrows), and the membrane distribution of active GSDMD (Figure 8C, white arrows). However, silica-induced NLRP3 inflammasome activation was significantly inhibited by MCC950 treatment. Notably, in the 3D culture system, the silica particles not only potently inhibited lung organoid development from lungospheres (Figures 8D and 8E) but also suppressed cell proliferation (as indicated by Ki67 immunostaining), which was alleviated by the NLRP3 inflammasome inhibitor MCC950 (Figure 8F). Moreover, p63, SOX2 and SOX9 immunofluorescence staining showed that silica treatment significantly decreased the proportion of p63/SOX2 or SOX9/SOX2 double-positive cells (Figures 8G-J) in the 3D organoid model, which was partially reversed by the NLRP3 inflammasome inhibitor MCC950.

## Discussion

Long-term exposure to crystalline silica particles leads to silicosis, a progressive fibrotic lung disease. Although silicosis is an old disease, our understanding of the disease pathogenesis is limited and no effective treatment is currently available. Over the past two decades, silica has been shown to be a potent activator of the NLRP3 inflammasome, which is a molecular platform mediating either the maturation of inflammatory factors (such as IL-1β and IL-18) or inflammatory cell death (pyroptosis) 31, 64, 65. Increasing evidence confirmed that NLRP3 inflammasome-mediated chronic inflammation participates in the development of silicosis 6. In animal models of silicosis, the NLRP3 inflammasome was persistently activated in the lung, while genetic or pharmacological inhibition of the NLRP3 inflammasome alleviated the inflammation and fibrosis associated with silicosis 6, 66. Once inhaled, silica particles deposit on the surface of distal airways and are difficult to remove or degrade, subsequently initiating a cycle of persistent inflammation and repeated lung epithelial injury. In lung macrophages and epithelial cells, silica triggered NLRP3 inflammasome-mediated inflammatory cascades and the secretion of IL-1β and IL-18, which further induced the production of other proinflammatory and profibrotic cytokines, such as TGF-β1, IL-6, and TNF-α 67, 68. Additionally, the silica-induced recurrent cycle of NLRP3 inflammasome activation in epithelial cells also led to the EMT, resulting in fibroblast differentiation and proliferation, as well as extracellular matrix deposition 31, 69. Moreover, persistent activation of the NLRP3 inflammasome caused pyroptosis, a specific form of programmed cell death 30, 39, which exacerbated the tissue damage and fibrosis. Therefore, the NLRP3 inflammasome has become a promising target for the prevention and treatment of silica-related lung injuries and diseases.

Although the NLRP3 inflammasome plays a major role in silica-driven inflammation and fibrosis, little is known about its effects on lung regeneration. Normal lung epithelial repair or regeneration is essential for restoring functions after lung injuries, while maladaptive or aberrant epithelial regeneration contributes to airway remodeling and fibrosis in chronic lung diseases, such as COPD and IPF 70. In the past decade, significant advances have been achieved in understanding the molecular and cellular mechanisms of tissue repair and regeneration with the advent of stem/progenitor cell-based organoid technologies 32. Regarding lung organoid studies, 2D ALI and 3D organoid models of lung epithelial stem/progenitor cells are widely used due to their multidimensional niche, which mimics the morphological and cellular compositions of the airway epithelium *in vivo* 35. In our study, 2D and 3D organotypic models using mouse LSPCs were successfully established, based on the results of morphological analyses (Figures S1-S3). Moreover, our organotypic systems recapitulated the architectural and functional properties of the airway epithelium *in vivo*, including mucous production and the movement of mucus globules across the epithelial surface by cilia (Videos S1-S3). By using these organotypic models, the role of the NLRP3 inflammasome in silica-induced lung epithelial injury, repair and regeneration was investigated for the first time. Our results showed that silica constantly activated the NLRP3 inflammasome in both 2D and 3D organotypic models.

In the 2D ALI system, silica acting on the apical surface of the LSPC-derived airway epithelium, which mimicked silica deposition in the distal airway, not only induced inflammatory cell death through pyroptosis but also increased cell proliferation. The disorganization and hyperplasia of silica-treated airway epithelium indicated that silica caused an imbalance between cell death and regeneration. In another words, although silica induced cell death, it exerted more potent effects on facilitating cell proliferation in the 2D ALI system. This opinion was supported by the results from the EdU incorporation proliferation assay. Although the mechanism remains unclear, a reasonable hypothesis is that, by activating the NLRP3 inflammasome, silica potentially creates an inflammatory niche enriched with IL-1β and TNF-α to facilitate epithelial progenitor proliferation 71. Additionally, increased ACT and mucin expressions in the LSPC-derived airway epithelium suggested that silica enhanced mucociliary differentiation and mucus production in the 2D ALI system. Notably, although the silica treatment increased the number of ciliated cells, the ultrastructure and dynamics of cilia were damaged. These morphological and functional abnormalities of the LSPC-derived airway epithelium induced by silica were restored by the selective NLRP3 inflammasome inhibitor MCC950. Therefore, these findings from the 2D ALI system suggest that the NLRP3 inflammasome might be a key regulator of either the balance between cell injury and regeneration or mucociliary function of the airway epithelium under the stress of silica.

Mucus overproduction and mucociliary dysfunction of the airway epithelium are involved in the pathogenesis of chronic lung diseases such as COPD and fibrosis 72, 73. Increased mucin production occurs in response to various inflammatory cytokines, including IL-1β, IL-6, IL-9, IL-13, and TNF-α 74-76, and excessive mucus aggregation in the distal airways leads to recurrent injury/inflammation/repair cycles with impaired mucus clearance and mucosal host defense 50, 77. During the repair of the injured mucociliary epithelium in the ALI system, silica-induced NLRP3 inflammasome activation led to either MUC5AC or MUC5B overproduction, multiciliogenesis, and ciliary disorientation, structural defects and dyskinesia. Based on accumulating evidence, excessive mucus production presents a challenge for the function of ciliated epithelial cells. MUC5AC is produced mainly in superficial airway goblet cells with pathological roles in the progression of airway hyperresponsiveness, mucous metaplasia and airway mucus plugging 78, 79. MUC5B is predominantly expressed in submucosal glands and performs physiological functions to clear inhaled particulates or pathogens 79, 80. It is also upregulated in primary ciliary dyskinesia 74, 81, and its overexpression results in airway epithelial injury and fibrillar collagen accumulation that expands and displaces normal lung tissue 50, 74, 77. In patients with cystic fibrosis, mucus accumulation and dehydration on the surface of the airway epithelium increased the concentration and viscosity of epithelial lining fluid, resulting in the collapse and reduced motility of cilia 73. Overexpression of MUC5B in bronchoalveolar epithelium directly impairs mucociliary clearance, which plays a causative role in bleomycin-induced pulmonary fibrosis 73. More recently, one study using the ALI culture of human bronchial epithelial cells and a mouse model of lung fibrosis induced by bleomycin reported that MUC5B overproduction led to cilia gene expression in IPF airway epithelial cells, which subsequently enhanced multiciliogenesis, as evidenced by both the number of ciliated cells and the disrupted motile cilia structure 50. Notably, the administration of one dose of the cytotoxic agent bleomycin was sufficient to initiate the long-term activation of the NLRP3 inflammasome in the mouse lung 82. In the present study, we found that silica treatment also caused MUC5AC/MUC5B hypersecretion and mucociliary dysfunction during the differentiation of LSPCs into the airway epithelium in the 2D ALI system. However, these silica-induced structural and functional abnormalities in the airway epithelium were reversed by pharmacological inhibition of the NLRP3 inflammasome. Together, these findings suggest that, in fibrotic lung diseases of different etiologies, the release of inflammatory factors mediated by the activated NLRP3 inflammasome might be an upstream event driving mucus hypersecretion and cell death. Thereafter, the changes in cytokines and matrix of the extracellular niche trigger aberrant airway epithelial regeneration from basal progenitors, including abnormal proliferation and differentiation, which finally results in distal airway epithelium remodeling.

During normal development, lung epithelium originates from multipotent progenitors expressing the transcription factor SOX9 (SOX9^+^), which undergo branching morphogenesis to build the respiratory tree and differentiate into daughter cells that give rise to both proximal air-conducting airways and distal gas-exchanging alveoli 83-85. On the one hand, SOX9^+^ progenitors directly differentiate into the major lung epithelial cell types in either conducting airways or alveoli in the injured lungs of adult mice 86. On the other hand, SOX9^+^ progenitors differentiate into SOX2^+^ daughter cells to establish proximal conducting airways with specific SOX9^+^/SOX2^+^ proximodistal patterns as branching proceeds 48, 87. SOX2 is another important transcription factor responsible for the emergence of basal cells and bronchoalveolar stem cells in the lung 88. In the airway epithelium, SOX2 not only facilitates the expression of basal cell-specific transcription factor gene p63 to generate the basal cell lineage 18, 88 but also drives basal cells into a hyperproliferative state to renew the mucociliary epithelium after injuries 52, 89. The specific spatiotemporal distribution of SOX9 and SOX2 contributes to proper cell proliferation and differentiation, allowing lung development, repair and regeneration. In response to injury caused by smoking, SOX9 drives the generation of airway basal cells and leads to the repair of the mucociliary epithelium 52, 53. In bleomycin-induced lung injury, SOX2 regulates alveolar epithelial plasticity by inducing the emergence of progenitor-like cells, but continuous proliferation of SOX2^+^ cells disrupts alveolar structure, including septal rupture 90. In addition to its effects on cell proliferation, SOX2 is also essential for the differentiation of basal cells into ciliated and secretory cells 91. In our research, SOX9 expression was upregulated and the proportion of SOX9^+^/SOX2^+^ cells was increased in silica-treated ALI cultures, which contributed to the hyperproliferation and abnormal differentiation of the LSPC-derived airway epithelium. Nevertheless, the SOX9- and SOX2-mediated silica-induced hyperplasia of the airway epithelium was effectively ameliorated by NLRP3 inflammasome inhibition, indicating that NLRP3 inflammasome activation might be a central event in aberrant epithelial regeneration observed in this injury model. Although evidence for the direct regulatory effects of the NLRP3 inflammasome on the expression of SOX superfamily genes is lacking, the inflammatory microenvironment induced by the activated NLRP3 inflammasome might alter the development-related signals in the LSPC niche to modulate subsequent regeneration.

The epithelial stem/progenitor cell niche is composed of supporting cells, extracellular matrix, and various growth signaling molecules that orchestrate normal development or regeneration following injury. Accumulating evidence indicates that NF-κB is an important inflammatory signal that not only mediates the expression of the NLRP3 inflammasome components but also increases the production of cytokines (such as IL-1β) induced by the NLRP3 inflammasome 55, 92. Although increased activity of the NF-κB signal promoted inflammatory responses to repair the epithelium during acute damage, its continuous upregulation created a chronic inflammatory microenvironment, leading to LSPC dysfunction including pyroptotic cell death, EMT and abnormal mucociliary differentiation. Notably, the activation of NF-κB directly promotes the transcription of vital targeted genes in different cells including Shh and Wnt 93-96. The Shh-Gli and Wnt/β-catenin pathways are two lung development-related signals and involved in maintaining the quiescence of progenitor cells and regenerative responses to injury 58, 97, 98. During the repair of lung injury, Shh-Gli signaling promotes cell proliferation 99, but the lack of this signaling leads to aberrant epithelial repair and regeneration 58, 100. Similarly, Wnt/β-catenin signaling is another pathway that is critical for lung homeostasis and regeneration by promoting the function and behavior of LSPCs. Following tracheal damage, canonical Wnt/β-catenin signaling is activated within basal cells to regenerate the airway epithelium, and the constitutively active β-catenin protein drives the generation of ciliated cells 97. Previous reports showed that SOX9 is a downstream target of canonical Wnt/β-catenin signaling, and its activation alters the spatial expression of SOX9 and SOX2 in developing airways 101. Furthermore, Wnt10a is a ligand that binds with the Fzd/LRP receptor to activate downstream β-catenin in the canonical Wnt/β-catenin signaling pathway, and its overexpression in lung-resident mesenchymal stem cells was correlated with Shh-Gli signaling activation 60, indicating crosstalk between the Shh-Gli and Wnt/β-catenin signaling pathways. In our silica-treated ALI cultures from LSPCs, key molecules, including receptor ligands and downstream signaling molecules, in the Shh-Gli and Wnt/β-catenin signaling pathways were significantly upregulated, which were efficiently inhibited by treatment with the NLRP3 inflammasome inhibitor MCC950. This result indicated that in addition to initiating inflammation, NLRP3 inflammasome activation also participated in the regulation of development- and regeneration-associated signals. Based on the significant role of the two pathways in regeneration, we believe that these reactivated pathways drove LSPC proliferation and differentiation to repair injured tissue and reestablish structural integrity. However, silica-induced sustained activation of the NLRP3 inflammasome caused continuous inflammatory responses, leading to persistent exaggerated activation of the Shh-Gli and Wnt/β-catenin pathways, subsequent epithelial disorganization, multiciliogenesis, mucociliary dysfunction and basal cell ectopic distribution. These morphological and functional abnormalities were considered a consequence of aberrant regeneration stimulated by prolonged and chronic Shh-Gli and Wnt/β-catenin activity. Therefore, silica-induced NLRP3 inflammasome activation might enhance IL-1β-NF-κB signaling to subsequently promote the expression of ligands mediating the Shh and Wnt signals. Subsequently, overactivation of the Shh-Gli and Wnt/β-catenin pathways resulted in upregulation of the transcription factors SOX9/SOX2 and aberrant repair and regeneration of the LSPC-derived airway epithelium.

Interestingly, in the 3D organoid culture system, silica-induced NLRP3 inflammasome activation induced potent pyroptosis, reduced cell proliferation, and inhibited epithelial differentiation in lung organoids derived from lungospheres. Furthermore, sustained silica stimulation decreased the coexpression of SOX9/SOX2 in the organoids, and inhibited organoid morphogenesis and epithelial regeneration. These results were different from the findings obtained from the LSPC-derived airway epithelium in 2D ALI cultures. Considering the action of silica on apical surface cells (ciliated and secretory cells) in the 2D model but on outside cells (basal cells or progenitors) in the 3D model, these differences between the two models suggest that the epithelial apical-basal polarity influences cell vulnerability to damage. Cell apical-basal polarity plays a vital role in lung epithelial homeostasis 102, as well as cellular behaviors such as migration and asymmetric division in shaping the complex 3D architecture 103. In the airway epithelium, epithelial cells have structurally and functionally distinct apical and basal faces 104, and distinct alterations in apical-basal polarity can dictate the differentiation of the airway epithelium 105. Specifically, tight junctions and cilia on the apical surface are crucial for the establishment and maintenance of the epithelial barrier 104, while the basal surface attached to the basement membrane is responsible for adhesion and signaling 106. Therefore, in the 2D and 3D models, silica stimulation of apical differentiated cells or basal stem/progenitor cells led to different responses and results. In the 2D ALI system, LSPCs differentiated along the vertical axis and were polarized in a single direction, and the apical surface of the epithelial cells was exposed to the atmosphere as well as silica particles during proliferation and differentiation. In this microenvironment, sustained stimulation with silica on the apical surface of the airway epithelium damaged upper layer differentiated progeny cells damage and subsequently induced compensatory hyperproliferation of basal cells or progenitors, resulting in dysregulated epithelial repair and airway remodeling with irreversible structural changes. However, in the 3D organoid model, the basal layer of lungosphere-derived organoid was polarized and oriented outward in the Matrigel. In this lumen-like structure, basal cells or progenitors were predominantly distributed in the outermost layer, while differentiated progeny cells were located inside the lumen of the organoids. Thus, once cocultured with silica in the 3D system, the outer progenitor cells in lungospheres were directly exposed to the injury caused by silica particle, which constantly activated NLRP3 inflammasome to induce continuous pyroptosis of progenitors. The loss of basal cells (p63^+^) and progenitors (SOX9^+^, SOX2^+^) reduced the number of proliferative cells and finally resulted in organoid formation failure. Based on the results from 2D ALI and 3D organoid systems, sustained NLRP3 inflammasome activation in cells from the apical layer or basal layer of the LSPC-derived airway epithelium produced different outcomes for epithelial regeneration, indicating that the NLRP3 inflammasome might play a complex but central role in lung epithelial repair and regeneration after silica-induced injuries.

## Conclusions

Collectively, our data from LSPC-derived organotypic models suggest that the NLRP3 inflammasome plays a vital role in airway epithelial injury, repair and regeneration. Pharmacological blockade of continuous NLRP3 inflammasome activation might reduce excessive epithelial cell death and prevent aberrant lung epithelial repair and regeneration induced by silica. The NLRP3 inflammasome potentially represents a promising therapeutic target for inhalable particle-related lung injuries and diseases.

## Materials and methods

### Reagents

Crystalline silica (CAS14808-60-7; purity 99%; particle diameter 0.5-10 μm) was purchased from Sigma‒Aldrich. The silica particles were incubated at 180°C for 2 h to inactivate endotoxins and remove water and were then suspended in 0.9% sterile saline to a concentration of 10 mg/ml. The silica suspension was sonicated for 20 min and mixed thoroughly prior to use.

The NLRP3 inflammasome selective inhibitor MCC950 (CAS256373-96-3) was purchased from Selleck Chemicals and dissolved in sterile normal saline to prepare a 10 mM stock solution. MCC950 was added 30 min prior to silica stimulation.

### Mice

Lung tissues were obtained from neonatal C57BL/6 mice. All procedures involving animals were approved by the Institutional Animal Care and Use Committee of Nanjing Medical University (NJMU/IACUC-2012034).

### Lungosphere formation assays 107

Peripheral lung tissue without the trachea and bronchi was mechanically dissociated with scalpels and digested by a solution of 2 mg/ml collagenase A, 2 mg/ml trypsin, and 50 μg/ml gentamicin (Sigma) in DMEM/F-12 (Gibco) for 45 min at 37°C with shaking. The digestion was stopped by the addition of 10% (v/v) fetal bovine serum (Gibco). The cell suspension was filtrated by a 100 μm cell strainer (Falcon) to eliminate tissue debris and cell clumps and was then collected by centrifugation at 4°C for 10 min. After being washed with DMEM/F-12 and undergoing centrifugation, the cell pellet was treated with red blood cell lysis buffer (Beyotime) 3 times for 2 min each. The cells were centrifuged again and resuspended in 20 U/ml DNase I (Sigma). After a final wash with DMEM/F-12, the cell suspension was filtrated through a 40 μm cell strainer (Falcon) and then seeded into nonadherent 25 cm^2^ flasks pretreated with poly-HEMA (Sigma) in lungosphere medium [1×B-27, 100 U/ml penicillin, 100 μg/ml streptomycin (Gibco), 4 μg/ml heparin (STEMCELL), 20 ng/ml murine epidermal growth factor (mEGF; Peprotech), 10 ng/ml murine fibroblast growth factor 2 (mFGF2; Peprotech), and 10 μM Y-27632 (STEMCELL) in DMEM/F-12] (1.0×10^6^ cells/ml). The flasks were incubated in a humidified atmosphere at 37°C and 5% CO_2_, and half the volume of medium was changed every 2 to 3 days.

### Airway epithelium-like structures at the air-liquid interface (ALI)

The lungospheres were washed with DMEM/F-12 and resuspended in TrypLE™ Express (Gibco). After incubation for 5-10 min at 37°C, the lungospheres were gently pipetted to generate single-cell suspensions. The cell suspension was passed through a 40 μm cell strainer to remove undigested cell clumps. The resulting single cells were again centrifuged and resuspended in PneumaCult™-Ex Plus Medium (STEMCELL) supplemented with 20 ng/ml mEGF. Transwell inserts (Corning) were seeded with 1.0×10^5^ cells in 0.5 ml medium per insert (apical chamber), and 1 ml medium was added to one well of the 12-well plate (basal chamber). The plates were incubated in a humidified atmosphere at 37°C and 5% CO_2_, and fresh culturing medium changes were performed on both sides of the inserts every 2 days using PneumaCult™-Ex Plus Medium. Once confluence was achieved in the submerged growth phase, the ALI culture was initiated by gently aspirating the medium from both the basal and apical chambers and adding only 1 ml of PneumaCult™-ALI Medium (STEMCELL) (supplemented with 20 ng/ml mEGF) to the basal compartment 108. Silica suspension was added to the apical chamber at the same time, and the remaining liquid was removed after settling, thereby exposing the luminal surface to the atmosphere (day 1 post-ALI). The medium in the basal chamber was changed with fresh medium every 2 days until day 28 post-ALI, leaving the apical chamber empty.

### Lung organoids in Matrigel

The lungospheres were collected from the nonadherent flasks, washed with DMEM/F-12, and then mixed with Growth Factor Reduced Matrigel (Corning) and silica particles. The Matrigel-silica-lungosphere mixture was pipetted into a 24-well plate in domes and incubated at 37°C for 30 min for solidification. PneumaCult™-ALI Medium supplemented with 20 ng/ml mEGF was added to each well. The plate was incubated in a cell culture incubator (37°C, 5% CO_2_) in which the medium was replaced with fresh medium every 3 days.

### Histological, immunohistochemical and immunofluorescence analyses

Lung organoids harvested from Matrigel by Cell Recovery Solution (Corning) and ALI cultures were fixed with 4% (w/v) paraformaldehyde for 20 min and washed with phosphate buffer solution (PBS). Then, the samples were processed via a standard procedure for paraffin and optimal cutting temperature compound (SAKURA) embedding. Paraffin or frozen sections were cut into 5 and 15 μm thicknesses, respectively. For histological staining, paraffin sections were stained using standard protocols for hematoxylin and eosin (H&E), periodic acid-Schiff (PAS) and Masson's trichrome staining reagents. For the immunohistochemistry studies, paraffin sections were cleared twice with xylene and rehydrated. Microwave antigen repair was carried out at 95°C for 20 min. The slides were blocked with QuickBlock™ Blocking Buffer (Beyotime) for 30 min at room temperature and then incubated at 4°C overnight with NLRP3 (Novus, 1:50), Caspase-1 (Abcam, 1:100) and IL-1β (Abcam, 1:200) antibodies. Subsequently, the sections were incubated with secondary antibodies followed by avidin-biotin solution (Vector Laboratories). The chromogen was developed with DAB (3,3'-diaminobenzidine) (Vector Laboratories) for visualization. For the immunofluorescence analysis, incubations were performed overnight at 4°C with the following primary antibodies diluted as indicated: rabbit anti-GSDMD antibody (Affinity) 1:400, mouse anti-acetylated tubulin (ACT) antibody (Proteintech) 1:200, rabbit anti-MUC5AC antibody (Absin) 1:100, mouse anti-MUC5B antibody (Abcam) 1:200, rabbit anti-ZO-1 antibody (Proteintech) 1:200, mouse anti-Vimentin antibody (Abcam) 1:200, rabbit anti-p63 antibody (GeneTex) 1:1000, rabbit anti-p63 antibody (Abcam) 1:200, rat anti-p75 NGF Receptor antibody (Abcam) 1:200, rat anti-SOX2 antibody (Invitrogen) 1:200, rabbit anti-SOX9 antibody (Abcam) 1:400, rabbit anti-ASC antibody (Cell Signaling Technology) 1:800, and rabbit anti-Ki67 antibody (Affinity) 1:200. The sections were washed with PBST and incubated with goat anti-mouse-Alexa 488 (Invitrogen), goat anti-rabbit-Alexa 555 (Invitrogen) and goat anti-rat-Alexa 647 (Abcam) at a 1:1000 dilution in PBST for 1 h at room temperature and then counterstained with DAPI (Beyotime) for 10 min. For the x-y plane and 3D reconstruction observations, the Transwell membranes were removed from the plastic support and placed onto glass slides. All samples were covered with ProLong™ Gold antifade reagent (ThermoFisher Scientific). Images were captured using a Leica Stellaris STED, Zeiss LSM710 confocal microscope, Leica Thunder DMi8 Imager or Olympus VS200 slide scanner.

### Proliferation assay

After stimulation with 50 μg/cm^2^ silica for 28 days, the polyester (PET) membranes of Transwell inserts were removed for further research. To assess cell proliferation in ALI cultures, we used a 5-ethynyl-2'-deoxyuridine (EdU) detection kit (RiboBio). Fluorescent slides were observed on a Leica Thunder DMi8 microscope. Three duplicates were performed for each group, and twenty randomly selected fields (40× objective) were analyzed to calculate the proportion of positive cells.

### Western blot analysis

Total proteins of ALI cultures were extracted with RIPA buffer (ThermoFisher Scientific) containing protease and phosphatase inhibitor cocktail (Roche) for western blot. Equal protein samples (40 μg) were subjected to electrophoresis in 8-12% SDS‒PAGE gels and subsequently transferred onto PVDF membranes (Millipore). The blots were blocked and then incubated overnight at 4°C with the following primary antibodies: rabbit polyclonal antibodies against NLRP3 (1:500, Novus), Caspase-1 p20 (1:1000, Proteintech), GSDMD (1:1000, Affinity), ZO-1 (1:2000, Proteintech), p63 (1:1000, GeneTex), SOX2 (1:1000, Abcam), IκBα (1:1000, Cell Signaling Technology), Smo (1:1000, Abcam), Gli1 (1:2000, Novus), Wnt10a (1:1000, Abcam), p-GSK-3β (1:1000, Cell Signaling Technology), β-actin (1:2000, Proteintech), and GAPDH (1:2000, Proteintech); rabbit monoclonal antibodies against pro-Caspase-1 (1:1000, Cell Signaling Technology), ASC (1:1000, Cell Signaling Technology), SOX9 (1:1000, Cell Signaling Technology), p-IκBα (1:1000, Cell Signaling Technology), p-NF-κB p65 (1:1000, Cell Signaling Technology), NF-κB p65 (1:1000, Cell Signaling Technology), GSK-3β (1:1000, Cell Signaling Technology), and β-catenin (1:1000, Cell Signaling Technology); and mouse monoclonal antibodies against E-Cadherin (1:1000, Cell Signaling Technology), Vimentin (1:1000, Abcam), and Shh (1:500, OriGene). After incubation with horseradish peroxidase (HRP)-conjugated secondary antibodies (Jackson ImmunoResearch), the protein bands were detected using WesternBright Quantum (Advansta) and quantified by a ChemiDoc^TM^ XRS+ system with Image Lab software (Bio-Rad).

### Cilia beat frequency (CBF) evaluation

On day 28 post-ALI, five visual fields per group were randomly selected to obtain video recordings of beating cilia using a 40× objective coupled with a Nikon video camera (Ti-S) (MP4). According to these digital video files, the ciliaFA plugin in ImageJ (National Institutes of Health, NIH) was used to assess CBF on the epithelial surface of differentiated ALI cultures 43.

### Scanning electron microscopy (SEM)

The ALI cultures were washed in PBS and fixed in 2.5% (v/v) glutaraldehyde solution overnight at 4°C. They were then dehydrated through increasing concentrations of ethanol and incubated in hexamethyldisilazane before being placed in a desiccator overnight. The membranes were detached from the inserts and mounted on aluminum stubs. The samples were sputter coated with gold before analysis on a JEOL JSM-7900F scanning electron microscope.

### Transmission electron microscopy (TEM)

The samples were washed in PBS and fixed in 2.5% (v/v) glutaraldehyde solution overnight at 4°C. Then, they were postfixed in 1% (v/v) osmium tetroxide solution for 30 min at room temperature. The ALI cultures were dehydrated in a series of graded ethanol solutions and embedded in epoxy resin. They were cut into 100 nm thin sections on a Leica ultramicrotome. The sections were stained with lead citrate and uranyl acetate for TEM using a JEOL JEM-1400Flash microscope.

### Statistical analysis

All quantitative data from at least three independent experiments are presented as the mean ± standard error of the mean (SEM). SPSS 18.0 software (SPSS Inc.) was used to perform the statistical analyses. One-way ANOVA followed by Fisher's LSD test were used to determine statistically significant differences, and statistical significance was confirmed at p < 0.05.

## Supplementary Material

Supplementary figures and video legends.

Supplementary video 1.

Supplementary video 2.

Supplementary video 3.

## References

[1] De Rose V, Molloy K, Gohy S, Pilette C, Greene CM (2018). Airway Epithelium Dysfunction in Cystic Fibrosis and COPD. Mediators of inflammation.

[2] Bui LT, Winters NI, Chung MI, Joseph C, Gutierrez AJ, Habermann AC (2021). Chronic lung diseases are associated with gene expression programs favoring SARS-CoV-2 entry and severity. Nature communications.

[3] Racanelli AC, Kikkers SA, Choi AMK, Cloonan SM (2018). Autophagy and inflammation in chronic respiratory disease. Autophagy.

[4] Cullinan P, Muñoz X, Suojalehto H, Agius R, Jindal S, Sigsgaard T (2017). Occupational lung diseases: from old and novel exposures to effective preventive strategies. The Lancet Respiratory medicine.

[5] Rose C, Heinzerling A, Patel K, Sack C, Wolff J, Zell-Baran L (2019). Severe Silicosis in Engineered Stone Fabrication Workers - California, Colorado, Texas, and Washington, 2017-2019. MMWR Morbidity and mortality weekly report.

[6] Lam M, Mansell A, Tate MD (2022). Another One Fights the Dust: Targeting the NLRP3 Inflammasome for the Treatment of Silicosis. American journal of respiratory cell and molecular biology.

[7] Duan JX, Guan XX, Yang HH, Mei WX, Chen P, Tao JH (2021). Vasoactive intestinal peptide attenuates bleomycin-induced murine pulmonary fibrosis by inhibiting epithelial-mesenchymal transition: Restoring autophagy in alveolar epithelial cells. International immunopharmacology.

[8] Yu Q, Fu G, Lin H, Zhao Q, Liu Y, Zhou Y (2020). Influence of silica particles on mucociliary structure and MUC5B expression in airways of C57BL/6 mice. Experimental lung research.

[9] Li S, Li Y, Zhang Y, Li S, Zhang M, Jin F (2020). N-Acetyl-Seryl-Asparyl-Lysyl-Proline regulates lung renin angiotensin system to inhibit epithelial-mesenchymal transition in silicotic mice. Toxicology and applied pharmacology.

[10] Yang J, Wu S, Hu W, Yang D, Ma J, Cai Q (2022). Bmi1 signaling maintains the plasticity of airway epithelial progenitors in response to persistent silica exposures. Toxicology.

[11] Churg A, Wright JL (2003). Bronchiolitis caused by occupational and ambient atmospheric particles. Seminars in respiratory and critical care medicine.

[12] Ferreira TP, de Arantes AC, do Nascimento CV, Olsen PC, Trentin PG, Rocco PR (2013). IL-13 immunotoxin accelerates resolution of lung pathological changes triggered by silica particles in mice. Journal of immunology (Baltimore, Md: 1950).

[13] Zhao H, Wang Y, Qiu T, Liu W, Yao P (2020). Autophagy, an important therapeutic target for pulmonary fibrosis diseases. Clinica chimica acta; international journal of clinical chemistry.

[14] Suri GS, Kaur G, Jha CK, Tiwari M (2021). Understanding idiopathic pulmonary fibrosis - Clinical features, molecular mechanism and therapies. Experimental gerontology.

[15] Tao N, Li K, Liu J, Fan G, Sun T (2021). Liproxstatin-1 alleviates bleomycin-induced alveolar epithelial cells injury and mice pulmonary fibrosis via attenuating inflammation, reshaping redox equilibrium, and suppressing ROS/p53/α-SMA pathway. Biochemical and biophysical research communications.

[16] John AE, Joseph C, Jenkins G, Tatler AL (2021). COVID-19 and pulmonary fibrosis: A potential role for lung epithelial cells and fibroblasts. Immunological reviews.

[17] Alysandratos KD, Herriges MJ, Kotton DN (2021). Epithelial Stem and Progenitor Cells in Lung Repair and Regeneration. Annual review of physiology.

[18] Lin SC, Chou YT, Jiang SS, Chang JL, Chung CH, Kao YR (2016). Epigenetic Switch between SOX2 and SOX9 Regulates Cancer Cell Plasticity. Cancer research.

[19] O'Koren EG, Hogan BL, Gunn MD (2013). Loss of basal cells precedes bronchiolitis obliterans-like pathological changes in a murine model of chlorine gas inhalation. American journal of respiratory cell and molecular biology.

[20] Liu M, Ren D, Wu D, Zheng J, Tu W (2015). Stem Cell and Idiopathic Pulmonary Fibrosis: Mechanisms and Treatment. Current stem cell research & therapy.

[21] Shi Y, Dong M, Zhou Y, Li W, Gao Y, Han L (2019). Distal airway stem cells ameliorate bleomycin-induced pulmonary fibrosis in mice. Stem cell research & therapy.

[22] Swanson KV, Deng M, Ting JP (2019). The NLRP3 inflammasome: molecular activation and regulation to therapeutics. Nature reviews Immunology.

[23] Gong T, Yang Y, Jin T, Jiang W, Zhou R (2018). Orchestration of NLRP3 Inflammasome Activation by Ion Fluxes. Trends in immunology.

[24] Theofani E, Semitekolou M, Samitas K, Mais A, Galani IE, Triantafyllia V (2022). TFEB signaling attenuates NLRP3-driven inflammatory responses in severe asthma. Allergy.

[25] Huppertz C, Jäger B, Wieczorek G, Engelhard P, Oliver SJ, Bauernfeind FG (2020). The NLRP3 inflammasome pathway is activated in sarcoidosis and involved in granuloma formation. The European respiratory journal.

[26] McElvaney OJ, Zaslona Z, Becker-Flegler K, Palsson-McDermott EM, Boland F, Gunaratnam C (2019). Specific Inhibition of the NLRP3 Inflammasome as an Antiinflammatory Strategy in Cystic Fibrosis. American journal of respiratory and critical care medicine.

[27] Wang X, Chen S, Zhao L, Shi X (2021). Protective effect of combination of anakinra and MCC950 against acute lung injury is achieved through suppression of the NF-κB-mediated-MAPK and NLRP3-caspase pathways. International immunopharmacology.

[28] Pan P, Shen M, Yu Z, Ge W, Chen K, Tian M (2021). SARS-CoV-2 N protein promotes NLRP3 inflammasome activation to induce hyperinflammation. Nature communications.

[29] Zeng J, Xie X, Feng XL, Xu L, Han JB, Yu D (2022). Specific inhibition of the NLRP3 inflammasome suppresses immune overactivation and alleviates COVID-19 like pathology in mice. EBioMedicine.

[30] Song M, Wang J, Sun Y, Pang J, Li X, Liu Y (2022). Inhibition of gasdermin D-dependent pyroptosis attenuates the progression of silica-induced pulmonary inflammation and fibrosis. Acta pharmaceutica Sinica B.

[31] Li X, Yan X, Wang Y, Wang J, Zhou F, Wang H (2018). NLRP3 inflammasome inhibition attenuates silica-induced epithelial to mesenchymal transition (EMT) in human bronchial epithelial cells. Experimental cell research.

[32] Kong J, Wen S, Cao W, Yue P, Xu X, Zhang Y (2021). Lung organoids, useful tools for investigating epithelial repair after lung injury. Stem cell research & therapy.

[33] Han Y, Duan X, Yang L, Nilsson-Payant BE, Wang P, Duan F (2021). Identification of SARS-CoV-2 inhibitors using lung and colonic organoids. Nature.

[34] Djidrovski I, Georgiou M, Tasinato E, Leonard MO, Van den Bor J, Lako M (2022). Direct transcriptomic comparison of xenobiotic metabolism and toxicity pathway induction of airway epithelium models at an air-liquid interface generated from induced pluripotent stem cells and primary bronchial epithelial cells. Cell biology and toxicology.

[35] Laube M, Pietsch S, Pannicke T, Thome UH, Fabian C (2021). Development and Functional Characterization of Fetal Lung Organoids. Frontiers in medicine.

[36] Salahudeen AA, Choi SS, Rustagi A, Zhu J, van Unen V, de la OS (2020). Progenitor identification and SARS-CoV-2 infection in human distal lung organoids. Nature.

[37] Xu G, Li Y, Zhang S, Peng H, Wang Y, Li D (2021). SARS-CoV-2 promotes RIPK1 activation to facilitate viral propagation. Cell research.

[38] Peng L, Wen L, Shi QF, Gao F, Huang B, Meng J (2020). Scutellarin ameliorates pulmonary fibrosis through inhibiting NF-κB/NLRP3-mediated epithelial-mesenchymal transition and inflammation. Cell death & disease.

[39] Burdette BE, Esparza AN, Zhu H, Wang S (2021). Gasdermin D in pyroptosis. Acta pharmaceutica Sinica B.

[40] Lachowicz-Scroggins ME, Yuan S, Kerr SC, Dunican EM, Yu M, Carrington SD (2016). Abnormalities in MUC5AC and MUC5B Protein in Airway Mucus in Asthma. American journal of respiratory and critical care medicine.

[41] Mao H, Wang Y, Yuan W, Wong LB (2009). Ciliogenesis in cryopreserved mammalian tracheal epithelial cells cultured at the air-liquid interface. Cryobiology.

[42] Konishi S, Gotoh S, Tateishi K, Yamamoto Y, Korogi Y, Nagasaki T (2016). Directed Induction of Functional Multi-ciliated Cells in Proximal Airway Epithelial Spheroids from Human Pluripotent Stem Cells. Stem cell reports.

[43] Smith CM, Djakow J, Free RC, Djakow P, Lonnen R, Williams G (2012). ciliaFA: a research tool for automated, high-throughput measurement of ciliary beat frequency using freely available software. Cilia.

[44] Guo H, Jian Z, Liu H, Cui H, Deng H, Fang J (2021). TGF-β1-induced EMT activation via both Smad-dependent and MAPK signaling pathways in Cu-induced pulmonary fibrosis. Toxicology and applied pharmacology.

[45] Qi Y, Zhao A, Yang P, Jin L, Hao C (2020). miR-34a-5p Attenuates EMT through targeting SMAD4 in silica-induced pulmonary fibrosis. Journal of cellular and molecular medicine.

[46] Hayes D Jr, Kopp BT, Hill CL, Lallier SW, Schwartz CM, Tadesse M (2019). Cell Therapy for Cystic Fibrosis Lung Disease: Regenerative Basal Cell Amplification. Stem cells translational medicine.

[47] Ma Q, Ma Y, Dai X, Ren T, Fu Y, Liu W (2018). Regeneration of functional alveoli by adult human SOX9(+) airway basal cell transplantation. Protein & cell.

[48] Gonçalves AN, Correia-Pinto J, Nogueira-Silva C (2020). ROBO2 signaling in lung development regulates SOX2/SOX9 balance, branching morphogenesis and is dysregulated in nitrofen-induced congenital diaphragmatic hernia. Respiratory research.

[49] Mahoney JE, Mori M, Szymaniak AD, Varelas X, Cardoso WV (2014). The hippo pathway effector Yap controls patterning and differentiation of airway epithelial progenitors. Developmental cell.

[50] Kim E, Mathai SK, Stancil IT, Ma X, Hernandez-Gutierrez A, Becerra JN (2022). Aberrant Multiciliogenesis in Idiopathic Pulmonary Fibrosis. American journal of respiratory cell and molecular biology.

[51] Whitsett JA, Haitchi HM, Maeda Y (2011). Intersections between pulmonary development and disease. American journal of respiratory and critical care medicine.

[52] Kim BR, Van de Laar E, Cabanero M, Tarumi S, Hasenoeder S, Wang D (2016). SOX2 and PI3K Cooperate to Induce and Stabilize a Squamous-Committed Stem Cell Injury State during Lung Squamous Cell Carcinoma Pathogenesis. PLoS biology.

[53] Ievlev V, Jensen-Cody CC, Lynch TJ, Pai AC, Park S, Shahin W (2022). Sox9 and Lef1 Regulate the Fate and Behavior of Airway Glandular Progenitors in Response to Injury. Stem cells (Dayton, Ohio).

[54] Chen J, Huang Y, Bian X, He Y (2022). Berberine Ameliorates Inflammation in Acute Lung Injury via NF-κB/Nlrp3 Signaling Pathway. Frontiers in nutrition.

[55] Wang Y, Liu F, Chen L, Fang C, Li S, Yuan S (2022). Neutrophil Extracellular Traps (NETs) Promote Non-Small Cell Lung Cancer Metastasis by Suppressing lncRNA MIR503HG to Activate the NF-κB/NLRP3 Inflammasome Pathway. Frontiers in immunology.

[56] Lau CI, Yánez DC, Papaioannou E, Ross S, Crompton T (2021). Sonic Hedgehog signalling in the regulation of barrier tissue homeostasis and inflammation. The FEBS journal.

[57] Aros CJ, Pantoja CJ, Gomperts BN (2021). Wnt signaling in lung development, regeneration, and disease progression. Communications biology.

[58] Chanda D, Otoupalova E, Smith SR, Volckaert T, De Langhe SP, Thannickal VJ (2019). Developmental pathways in the pathogenesis of lung fibrosis. Molecular aspects of medicine.

[59] Oda K, Yatera K, Izumi H, Ishimoto H, Yamada S, Nakao H (2016). Profibrotic role of WNT10A via TGF-β signaling in idiopathic pulmonary fibrosis. Respiratory research.

[60] Cao H, Chen X, Hou J, Wang C, Xiang Z, Shen Y (2020). The Shh/Gli signaling cascade regulates myofibroblastic activation of lung-resident mesenchymal stem cells via the modulation of Wnt10a expression during pulmonary fibrogenesis. Laboratory investigation; a journal of technical methods and pathology.

[61] Soriano-Teruel PM, García-Laínez G, Marco-Salvador M, Pardo J, Arias M, DeFord C (2021). Identification of an ASC oligomerization inhibitor for the treatment of inflammatory diseases. Cell death & disease.

[62] Zhang X, Liu Y, Deng G, Huang B, Kai G, Chen K (2021). A Purified Biflavonoid Extract From Selaginella moellendorffii Alleviates Gout Arthritis via NLRP3/ASC/Caspase-1 Axis Suppression. Frontiers in pharmacology.

[63] Kwak SB, Koppula S, In EJ, Sun X, Kim YK, Kim MK (2018). Artemisia Extract Suppresses NLRP3 and AIM2 Inflammasome Activation by Inhibition of ASC Phosphorylation. Mediators of inflammation.

[64] Campden RI, Warren AL, Greene CJ, Chiriboga JA, Arnold CR, Aggarwal D (2022). Extracellular cathepsin Z signals through the α(5) integrin and augments NLRP3 inflammasome activation. The Journal of biological chemistry.

[65] Yang Y, Wang H, Kouadir M, Song H, Shi F (2019). Recent advances in the mechanisms of NLRP3 inflammasome activation and its inhibitors. Cell death & disease.

[66] Song Z, Wang L, Cao Y, Liu Z, Zhang M, Zhang Z (2022). Isoandrographolide inhibits NLRP3 inflammasome activation and attenuates silicosis in mice. International immunopharmacology.

[67] Liu X, Wang J, Dou P, Zhang X, Ran X, Liu L (2021). The Ameliorative Effects of Arctiin and Arctigenin on the Oxidative Injury of Lung Induced by Silica via TLR-4/NLRP3/TGF-β Signaling Pathway. Oxidative medicine and cellular longevity.

[68] Yin H, Fang L, Wang L, Xia Y, Tian J, Ma L (2022). Acute Silica Exposure Triggers Pulmonary Inflammation Through Macrophage Pyroptosis: An Experimental Simulation. Frontiers in immunology.

[69] Li Y, Li M, Wang Y, Guan L, Liu X, Zeng M (2022). The interplay between ASMase signaling pathway and NLRP3 in the epithelial to mesenchymal transition of HBE cells induced by silica. Journal of applied toxicology: JAT.

[70] Barnes PJ, Anderson GP, Fagerås M, Belvisi MG (2021). Chronic lung diseases: prospects for regeneration and repair. European respiratory review: an official journal of the European Respiratory Society.

[71] Katsura H, Kobayashi Y, Tata PR, Hogan BLM (2019). IL-1 and TNFα Contribute to the Inflammatory Niche to Enhance Alveolar Regeneration. Stem cell reports.

[72] Ancel J, Belgacemi R, Diabasana Z, Perotin JM, Bonnomet A, Dewolf M (2021). Impaired Ciliary Beat Frequency and Ciliogenesis Alteration during Airway Epithelial Cell Differentiation in COPD. Diagnostics (Basel, Switzerland).

[73] Hancock LA, Hennessy CE, Solomon GM, Dobrinskikh E, Estrella A, Hara N (2018). Muc5b overexpression causes mucociliary dysfunction and enhances lung fibrosis in mice. Nature communications.

[74] Zhang L, Li Q, Liu Z, Wang Y, Zhao M (2019). The protective effects of bone mesenchymal stem cells on paraquat-induced acute lung injury via the muc5b and ERK/MAPK signaling pathways. American journal of translational research.

[75] Woo HJ, Yoo WJ, Bae CH, Song SY, Kim YW, Park SY (2010). Leptin up-regulates MUC5B expression in human airway epithelial cells via mitogen-activated protein kinase pathway. Experimental lung research.

[76] Turner J, Jones CE (2009). Regulation of mucin expression in respiratory diseases. Biochemical Society transactions.

[77] Dobrinskikh E, Estrella AM, Hennessy CE, Hara N, Schwarz MI, Kurche JS (2021). Genes, other than Muc5b, play a role in bleomycin-induced lung fibrosis. American journal of physiology Lung cellular and molecular physiology.

[78] Tajiri T, Matsumoto H, Jinnai M, Kanemitsu Y, Nagasaki T, Iwata T (2022). Pathophysiological relevance of sputum MUC5AC and MUC5B levels in patients with mild asthma. Allergology international: official journal of the Japanese Society of Allergology.

[79] Ma J, Rubin BK, Voynow JA (2018). Mucins, Mucus, and Goblet Cells. Chest.

[80] Jaramillo AM, Azzegagh Z, Tuvim MJ, Dickey BF (2018). Airway Mucin Secretion. Annals of the American Thoracic Society.

[81] Munye MM, Shoemark A, Hirst RA, Delhove JM, Sharp TV, McKay TR (2017). BMI-1 extends proliferative potential of human bronchial epithelial cells while retaining their mucociliary differentiation capacity. American journal of physiology Lung cellular and molecular physiology.

[82] Song C, He L, Zhang J, Ma H, Yuan X, Hu G (2016). Fluorofenidone attenuates pulmonary inflammation and fibrosis via inhibiting the activation of NALP3 inflammasome and IL-1β/IL-1R1/MyD88/NF-κB pathway. Journal of cellular and molecular medicine.

[83] Khattar D, Fernandes S, Snowball J, Guo M, Gillen MC, Jain SS (2022). PI3K signaling specifies proximal-distal fate by driving a developmental gene regulatory network in SOX9+ mouse lung progenitors. eLife.

[84] Li L, Feng J, Zhao S, Rong Z, Lin Y (2021). SOX9 inactivation affects the proliferation and differentiation of human lung organoids. Stem cell research & therapy.

[85] Rockich BE, Hrycaj SM, Shih HP, Nagy MS, Ferguson MA, Kopp JL (2013). Sox9 plays multiple roles in the lung epithelium during branching morphogenesis. Proceedings of the National Academy of Sciences of the United States of America.

[86] Nichane M, Javed A, Sivakamasundari V, Ganesan M, Ang LT, Kraus P (2017). Isolation and 3D expansion of multipotent Sox9(+) mouse lung progenitors. Nature methods.

[87] Ostrin EJ, Little DR, Gerner-Mauro KN, Sumner EA, Ríos-Corzo R, Ambrosio E (2018). β-Catenin maintains lung epithelial progenitors after lung specification. Development (Cambridge, England).

[88] Ochieng JK, Schilders K, Kool H, Boerema-De Munck A, Buscop-Van Kempen M, Gontan C (2014). Sox2 regulates the emergence of lung basal cells by directly activating the transcription of Trp63. American journal of respiratory cell and molecular biology.

[89] Tompkins DH, Besnard V, Lange AW, Keiser AR, Wert SE, Bruno MD (2011). Sox2 activates cell proliferation and differentiation in the respiratory epithelium. American journal of respiratory cell and molecular biology.

[90] Kapere Ochieng J, Schilders K, Kool H, Buscop-van Kempen M, Boerema-De Munck A, Grosveld F (2014). Differentiated type II pneumocytes can be reprogrammed by ectopic Sox2 expression. PloS one.

[91] Eenjes E, Buscop-van Kempen M, Boerema-de Munck A, Edel GG, Benthem F, de Kreij-de Bruin L (2021). SOX21 modulates SOX2-initiated differentiation of epithelial cells in the extrapulmonary airways. eLife.

[92] Zhong Z, Umemura A, Sanchez-Lopez E, Liang S, Shalapour S, Wong J (2016). NF-κB Restricts Inflammasome Activation via Elimination of Damaged Mitochondria. Cell.

[93] Vecchiotti D, Verzella D, Di Vito Nolfi M, D'Andrea D, Flati I, Di Francesco B (2022). Elevated NF-κB/SHh/GLI1 Signature Denotes a Worse Prognosis and Represent a Novel Potential Therapeutic Target in Advanced Prostate Cancer. Cells.

[94] Singh AP, Arora S, Bhardwaj A, Srivastava SK, Kadakia MP, Wang B (2012). CXCL12/CXCR4 protein signaling axis induces sonic hedgehog expression in pancreatic cancer cells via extracellular regulated kinase- and Akt kinase-mediated activation of nuclear factor κB: implications for bidirectional tumor-stromal interactions. The Journal of biological chemistry.

[95] Brandenburg J, Reiling N (2016). The Wnt Blows: On the Functional Role of Wnt Signaling in Mycobacterium tuberculosis Infection and Beyond. Frontiers in immunology.

[96] Lopez-Bergami P, Barbero G (2020). The emerging role of Wnt5a in the promotion of a pro-inflammatory and immunosuppressive tumor microenvironment. Cancer metastasis reviews.

[97] Raslan AA, Yoon JK (2020). WNT Signaling in Lung Repair and Regeneration. Molecules and cells.

[98] Villar J, Zhang H, Slutsky AS (2019). Lung Repair and Regeneration in ARDS: Role of PECAM1 and Wnt Signaling. Chest.

[99] Wang C, Cassandras M, Peng T (2019). The Role of Hedgehog Signaling in Adult Lung Regeneration and Maintenance. Journal of developmental biology.

[100] Peng T, Frank DB, Kadzik RS, Morley MP, Rathi KS, Wang T (2015). Hedgehog actively maintains adult lung quiescence and regulates repair and regeneration. Nature.

[101] Hashimoto S, Chen H, Que J, Brockway BL, Drake JA, Snyder JC (2012). β-Catenin-SOX2 signaling regulates the fate of developing airway epithelium. Journal of cell science.

[102] Buckley CE, St Johnston D (2022). Apical-basal polarity and the control of epithelial form and function. Nature reviews Molecular cell biology.

[103] Vladar EK, Königshoff M (2020). Noncanonical Wnt planar cell polarity signaling in lung development and disease. Biochemical Society transactions.

[104] Lu YZ, He XL, Liu F, Cheng PP, Liang LM, Wang M (2020). Bleomycin induced apical-basal polarity loss in alveolar epithelial cell contributes to experimental pulmonary fibrosis. Experimental cell research.

[105] Szymaniak AD, Mahoney JE, Cardoso WV, Varelas X (2015). Crumbs3-Mediated Polarity Directs Airway Epithelial Cell Fate through the Hippo Pathway Effector Yap. Developmental cell.

[106] Zajac AL, Horne-Badovinac S (2022). Kinesin-directed secretion of basement membrane proteins to a subdomain of the basolateral surface in Drosophila epithelial cells. Current biology: CB.

[107] Rabata A, Hampl A, Koledova Z (2017). Lungosphere Assay: 3D Culture of Lung Epithelial Stem/Progenitor Cells. Methods in molecular biology (Clifton, NJ).

[108] O'Boyle N, Sutherland E, Berry CC, Davies RL (2017). Temporal dynamics of ovine airway epithelial cell differentiation at an air-liquid interface. PloS one.

