# Supplementary material for: NLRP3 Inflammasome Mediates Silica-induced Lung Epithelial Injury and Aberrant Regeneration in Lung Stem/Progenitor Cell-derived Organotypic Models
Source: Int J Biol Sci. 2023 Mar 21;19(6):1875–93. doi: 10.7150/ijbs.80605 (PMC10092774; doi:10.7150/ijbs.80605)
Supplement: Supplementary file 1 — Supplementary figures and video legends. [file ijbsv19p1875s1.pdf]

Supplemental information

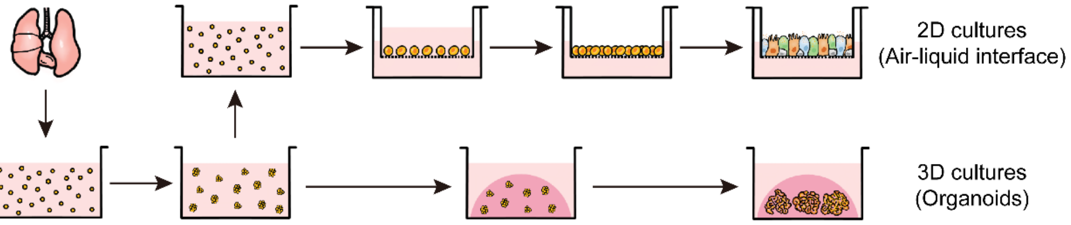

Figure S1

Generation of mouse airway epithelium-like structures (2D cultures) and lung organoids (3D cultures) from lungospheres.

Lungospheres were generated from disassociated lung stem/progenitor cells (LSPCs) of neonatal mice with serum-free medium in nonadherent conditions. These lungospheres were collected and cultured in 3D Matrigel domes, while lungosphere-disassociated LSPCs were cultured at the 2D air-liquid interface (ALI) to generate lung organoids or airway epithelium-like structures.

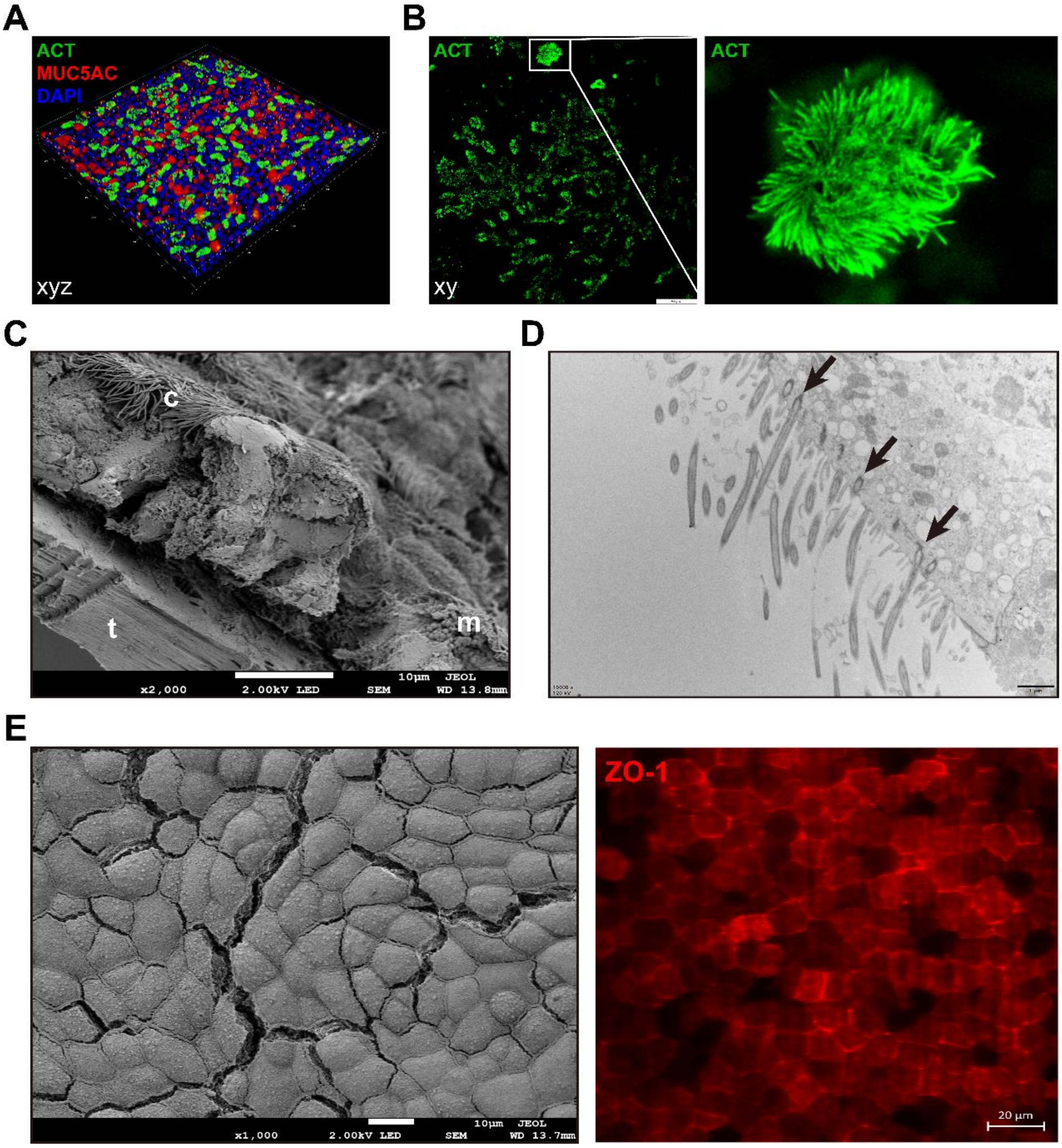

Figure S2

Identification of airway epithelium-like structures grown from LSPCs in the ALI model.

(A) 3D reconstruction (xyz plane) of the differentiated ALI cultures. ACT (green): cilia marker, MUC5AC (red): mucus-producing goblet cell marker.

(B) Representative photographs of the positive immunostaining of ACT in the x-y plane of differentiated ALI cultures. Scale bar, 50 μm.

(C) Cilia and mucus globules were observed on the apical surface of the LSPC-derived airway epithelium by scanning electron microscopy (SEM). (c), Cilia. (m), Mucus globules. (t), Transwell membrane. Scale bar, 10 μm.

(D) Longitudinal sections of mature cilia on the apical surface of the LSPC-derived airway epithelium showed the typical ultrastructure of basal bodies (black arrows) by transmission electron microscopy (TEM). Scale bar, 1 µm.

(E) Tight junctions on the apical surface of ALI cultures were confirmed by SEM and positive immunostaining of ZO-1. Scale bar, 10 µm and 20 µm.

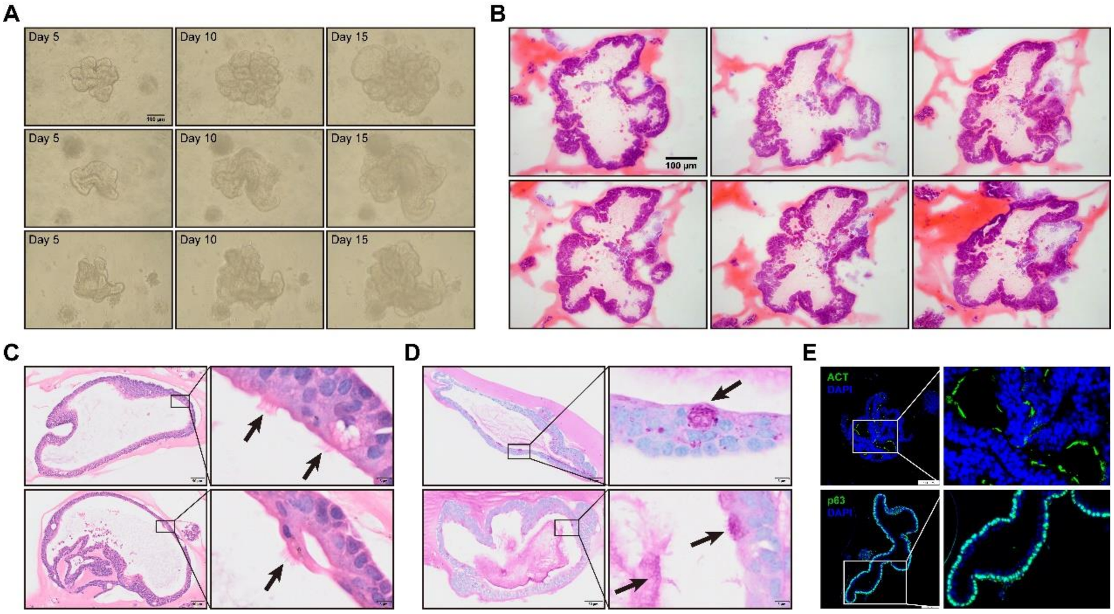

Figure S3

Development and identification of lung organoids grown from lungospheres in 3D Matrigel droplets.

(A) Growing branching morphogenesis of mouse lung organoids in 3D culture on days 5, 10 & 15. Scale bar, 100 µm.

(B) H&E staining of serial frozen sections revealed branching morphology and hollow lumen of lung organoids in the 3D culture. Scale bar, 100 µm.

(C) Representative photographs of H&E-stained paraffin sections confirmed that the lumen was surrounded by polarized airway epithelial cells, as well as the presence of cilia (black arrows) that pointed toward the lumen. Scale bars, 50 µm and 5 µm (magnified picture).

(D) Representative photographs of the PAS-stained paraffin sections. The black arrows indicate PAS-positive mucus secretion. Scale bars, 50 µm and 5 µm (magnified picture).

(E) Immunofluorescence characterization of frozen sections of lung organoids showed

ACT-positive ciliated cells distributed in the inner lumen, while p63-positive basal cells were located in the outer layer of the organoids. Scale bar, 100 μm.

Video S1

A video of functionally beating cilia was captured on day 28 post-ALI in 2D culture (400×).

Video S2

Differentiated ALI cultures possessed actively beating cilia that were capable of propelling mucus globules across the epithelial surface in 2D culture (400×).

Video S3

Vigorous beating cilia propelled mucus globules in the closed lumen of lung organoids in the 3D culture in a similar manner to that observed in the mucociliary escalator *in vivo* (400×).
